# Photophysical Properties and Protein Binding Studies of Piperazine-Substituted Anthracene-BODIPY Dyads for Antimicrobial Photodynamic Therapy

**DOI:** 10.3390/molecules30132727

**Published:** 2025-06-25

**Authors:** Stephen O’Sullivan, Leila Tabrizi, Kaja Turzańska, Ian P. Clark, Deirdre Fitzgerald-Hughes, Mary T. Pryce

**Affiliations:** 1 School of Chemical Sciences, Dublin City University, D09W6Y4 Dublin, Ireland; stephen.osullivan63@mail.dcu.ie (S.O.); leila.tabrizi@dcu.ie (L.T.); 2 Department of Clinical Microbiology, Royal College of Surgeons in Ireland, RCSI Education and Research, Beaumont Hospital, Beaumont, D09YD60 Dublin, Ireland; kajaturzanska20@rcsi.ie; 3 Central Laser Facility, Research Complex at Harwell, STFC Rutherford Appleton Laboratory, Harwell Campus, Didcot OX11 0QX, Oxfordshire, UK; ian.p.clark@stfc.ac.uk

**Keywords:** BODIPY, anthracene dyad, heavy-atom-free, photodynamic therapy, fluorescence, triplet state, antimicrobial activity

## Abstract

This work presents the synthesis, characterisation, photophysical properties, time-resolved spectroscopic behaviour, and biological evaluation of two structurally distinct heavy-atom-free BODIPY-anthracene dyads (**BDP-1**) and the newly designed 2,6-bis[1-(tert-butyl) 4-(prop-2-yn-1-yl) piperazine-1,4-dicarboxylate] BODIPY-anthracene (**BDP-2**), incorporating 2,6-alkynyl-piperazine substituents for potential application in antimicrobial photodynamic therapy. **BDP-1** exhibits absorption and emission maxima at 507 nm and 516 nm, respectively, with a Stokes shift of 344 cm^−1^ in dichloromethane (DCM), characteristic of unsubstituted BODIPYs. In contrast, **BDP-2** undergoes a red-shift in the absorption maximum to 552 nm (Stokes shift of 633 cm^−1^), which is attributed to the extended conjugation from the introduction of the alkyne groups. Time-resolved infrared spectroscopy confirmed efficient spin-orbit charge transfer intersystem crossing, and nanosecond transient absorption studies confirmed the formation of a long-lived triplet state for **BDP-2** (up to 138 µs in MeCN). A binding constant (K_b_) of 9.6 × 10^4^ M^−1^ was obtained for **BDP-2** when titrated with bovine serum albumin (BSA), which is higher than comparable BODIPY derivatives. **BDP-2** displayed improved hemocompatibility compared to **BDP-1** (<5% haemolysis of human erythrocytes up to 200 μg·mL^−1^). Antimicrobial activity of **BDP-1** and **BDP-2** was most potent when irradiated at 370 nm compared to the other wavelengths employed. However, **BDP-2** did not retain the potent (6 log) and rapid (within 15 min) eradication of *Staphylococcus aureus* achieved by **BDP-1** under irradiation at 370 nm. These findings demonstrate the rational design of **BDP-2** as a biocompatible, and heavy-atom-free BODIPY offering promise for targeted antimicrobial photodynamic therapeutic applications.

## 1. Introduction

The high prevalence and severity of infections involving multidrug-resistant (MDR) microbes in recent years is increasingly worrisome. Addressing antimicrobial resistance (AMR) effectively is a global priority given its potentially devastating consequences across healthcare systems in both the developed and developing world. It is predicted that by 2050, 10 million people will die from AMR-related illnesses annually if current trends continue. Developing alternative means of combating infection to conventional antibiotics is among the coordinated strategies being implemented by international agencies to address the AMR challenge [1,2]. Our over-reliance on conventional antibiotics, for which many pathogens have developed resistance, has accelerated their obsolescence. At the same time, the development of antibiotics with new modes of action has not progressed to market in Europe, with the exception of daptomycin in 2006.

Antimicrobial photodynamic therapy (aPDT) has emerged as a promising alternative for the treatment of bacteria-related infections, with an emphasis on open-wound infections [3,4,5,6,7,8]. While photodynamic therapy (PDT) was originally developed for cancer treatment, it has been adapted for treatment of microbial infections with potential for broad-spectrum applications [9,10]. Antibiotics such as penicillin, tetracycline, and vancomycin target specific pathways within the cell, whereas aPDT has a non-specific mode of action whereby a photosensitive molecule or photosensitiser absorbs light and uses that energy to convert oxygen into reactive oxygen species (ROS). These ROS can oxidise a variety of cellular components within the cell wall membranes in addition to nucleic acids, resulting in cell death. Singlet oxygen (^1^O_2_) is one ROS, formed by energy transfer (EnT) from the photosensitiser to triplet oxygen (^3^O_2_). As aPDT results in oxidative damage to multiple cellular targets, the development of resistance to this therapy is limited. However, ^1^O_2_ has a short lifetime in aqueous environments (~3.5 µs), which limits the area of effect by ROS due to the slow nature of diffusion [11]. This highlights the need for PSs that can effectively bind to or penetrate cells to maximise their effectiveness.

Here we described the development of boron dipyrromethene (BODIPY) dyes for aPDT. BODIPYs have been extensively studied in recent years for a wide range of disciplines, although they are primarily used as fluorescent tags due to their high fluorescence quantum yields (Φ_F_). BODIPYs typically display a λ_max_ at 500 nm corresponding to the S_0_ → S_1_ (π → π*) transition. While high fluorescence quantum yields are frequently reported in BODIPYs, this comes at the expense of a low triplet quantum yield (Φ_T_) and poor ROS generation. However, it is possible to promote spin-forbidden processes via intersystem crossing (ISC), with the most common approach incorporating heavy atoms such as Br, I, or transition metals [12]. While this may result in improved singlet oxygen quantum yields, heavy atoms are frequently associated with mammalian cell cytotoxicity [13,14]. A more appealing, though challenging, strategy involves enhancing ISC via structural modifications that increase molecular twisting or promote charge-transfer interactions. Recent studies have highlighted the role of spin-orbit charge-transfer intersystem crossing (SOCT-ISC) in orthogonal donor–acceptor systems, particularly in meso-anthracenyl-substituted BODIPYs [15,16]. These systems exploit conformational rigidity and orthogonality to decouple singlet and triplet surfaces, which can result in high quantum yields of the triplet state.

The demand for efficient, biocompatible photosensitisers for antimicrobial photodynamic therapy (aPDT) has spurred intense interest in heavy-atom-free (HAF) BODIPY systems. Among these, anthracene-BODIPY dyads have emerged as leading candidates due to their ability to populate triplet states through spin-orbit charge transfer intersystem crossing (SOCT-ISC), without the need for heavy atoms. Seminal studies by Filatov et al. and Wang et al. have established the fundamental role of orthogonal anthracene-BODIPY geometries in achieving high singlet oxygen quantum yields (Φ_Δ_) via efficient population of the triplet state [17,18,19]. Based on results from time-resolved electron paramagnetic resonance spectroscopy (TR-EPR) and DFT calculations, they proposed spin-orbit charge transfer as the dominating pathway for population of the triplet state in *meso*-substituted anthracenyl BODIPYs. This was attributed to the compact and orthogonal geometry, which resulted in efficient transitions from the singlet charge transfer state (CT_s_) to the first triplet excited state (T_1_). While the triplet quantum yields are low in non-polar solvents such as hexane and toluene, exceptionally high triplet quantum yields for HAF BODIPYs were reported in MeCN for **BDP-1** (Φ_T_ = 0.96 and Φ_Δ_ = 0.86) [19].

**Figure 1:**
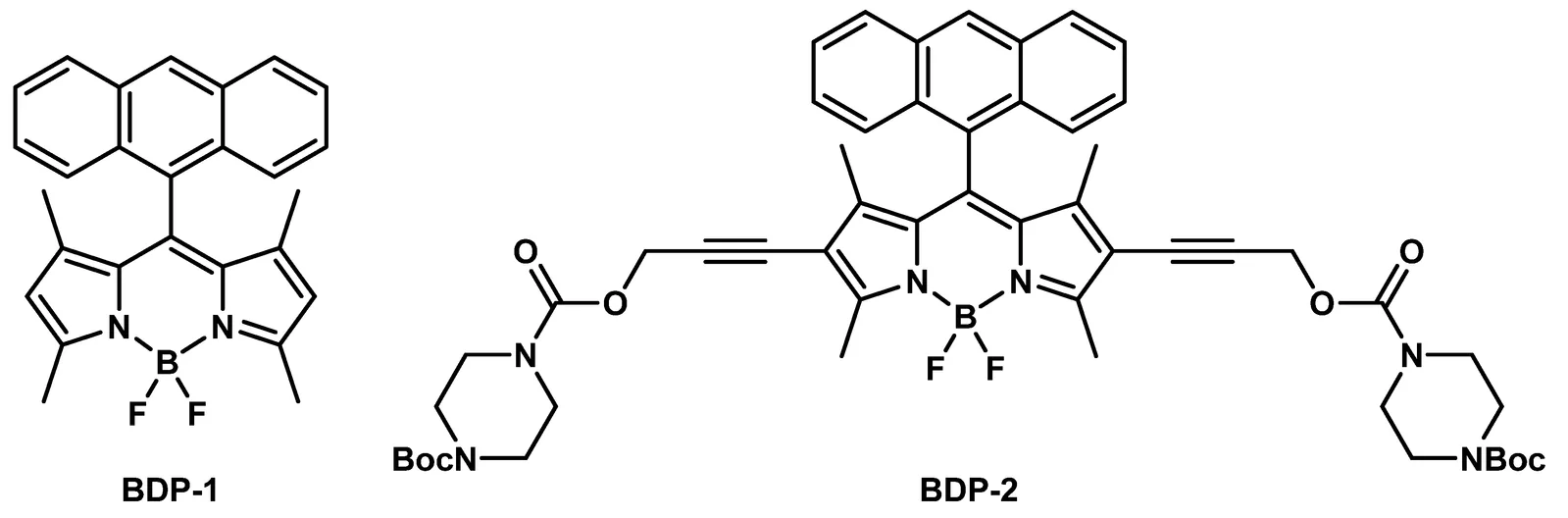
BODIPY structures in this work.

Building on these studies, we introduced a novel derivative (**BDP-2**, Figure 1) that integrates 2,6-alkynyl-piperazine substituents into the BODIPY core. This design enhances π-conjugation, facilitates protein interaction, and improves photophysical properties in polar environments—critical for biological applications. Unlike previously reported anthracene-BODIPY derivatives, **BDP-2** exhibits long triplet lifetimes, strong serum protein binding affinity, and excellent hemocompatibility, making it a promising candidate for biomedical applications. The piperazine moiety was incorporated as it is commonly a component in antibacterial drug scaffolds [20,21,22] to further enhance the biofunctional potential of this photosensitiser. Piperazine is a well-established pharmacophore known to improve aqueous solubility, cellular permeability, and protein-binding capacity, all of which are critical properties for effective photosensitiser performance in biological systems [23,24].

The suitability of photosensitisers for aPDT applications is influenced not only by optical and excited-state characteristics but also by their pharmacokinetic behaviour. Binding to serum proteins like bovine serum albumin (BSA) affects circulation time and bioavailability. The strong interaction of **BDP-2** with BSA, as evaluated by fluorescence quenching assays, highlights its therapeutic potential. Additionally, the red-shifted absorption and extended excited-state lifetimes of **BDP-2** improve its potential for biological targets [25,26,27,28,29].

This study focuses on the photophysical properties and biological evaluation of **BDP-1** and **BDP-2**, with an emphasis on the structural factors that govern triplet-state formation, protein interaction, and antimicrobial efficacy. These findings offer new insights into the rational design of BODIPY-based aPDT agents, thus providing a valuable framework for developing advanced, biocompatible photosensitisers.

## 2. Results and Discussion

**Scheme 1:**
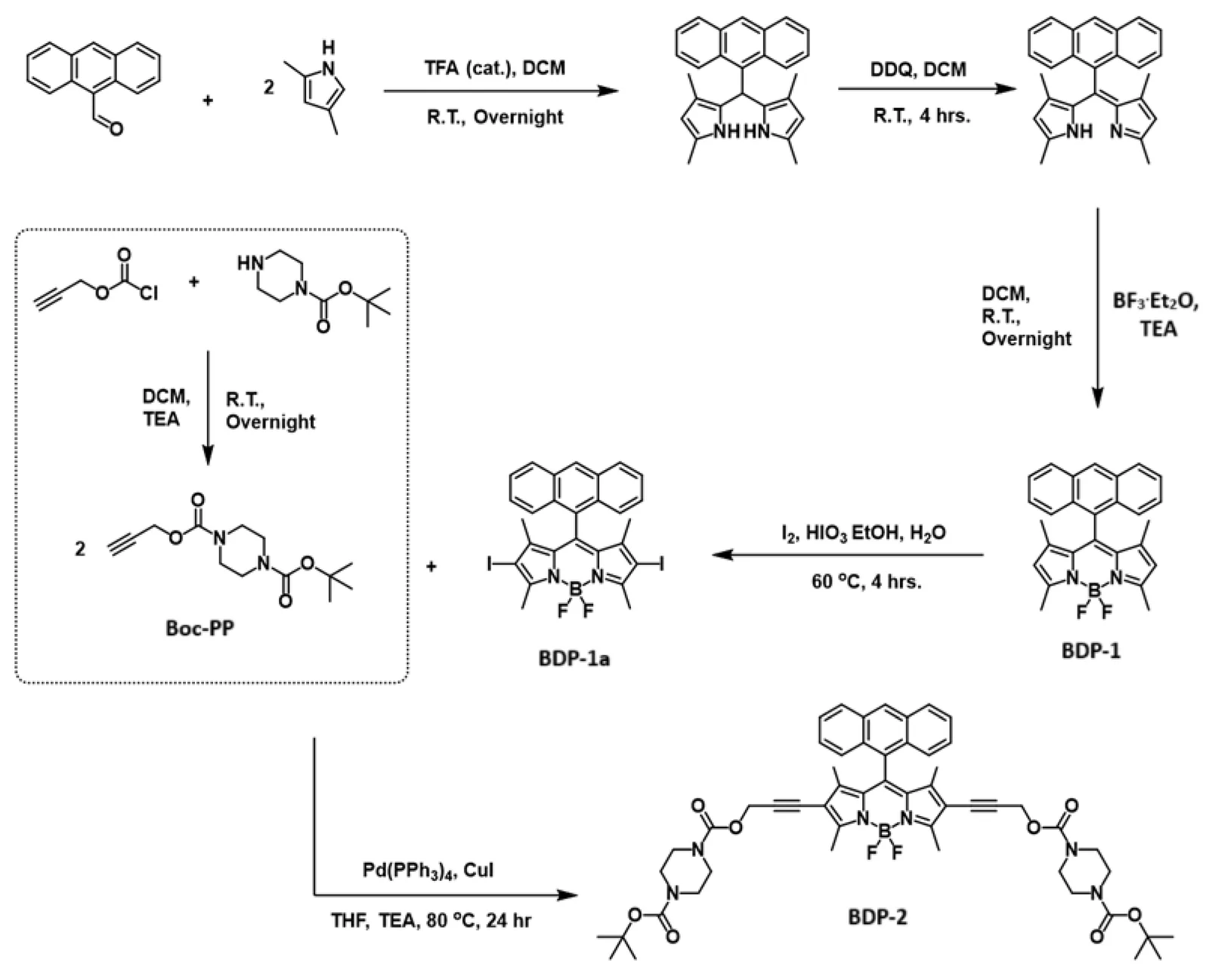
Synthetic pathway to **BDP-2**.

An overall scheme for the synthetic pathways employed in the preparation of the BODIPY compounds is displayed in Scheme 1. The synthesis of 1-(tert-butyl) 4-(prop-2-yn-1-yl) piperazine-1,4-dicarboxylate (**Boc-PP**) was achieved through the propargylation of Boc-piperazine using propargyl chloroformate, yielding a white solid in 94% yield without the need for further purification (Figure S5). In the final synthetic step, **BDP-2** was obtained via Sonogashira coupling of **BDP-1a** with **Boc-PP** in the presence of Pd(PPh_3_)_4_ and a CuI catalyst, producing a purple solid with a yield of 17%. The structure of **BDP-2** was supported by ^1^H NMR spectroscopy (Figure S6). Mass spectrometry further confirmed the product with a parent ion peak at *m*/*z* = 957.4471 for (M + H) ^+^ (Figure S10). The efficient iodination leading to **BDP-1a** facilitated the final coupling reaction, and the incorporation of the piperazine moiety into the BODIPY scaffold suggests potential for biological applications due to its biocompatibility and functional versatility.

### 2.1. Photophysical Properties

**Figure 2:**
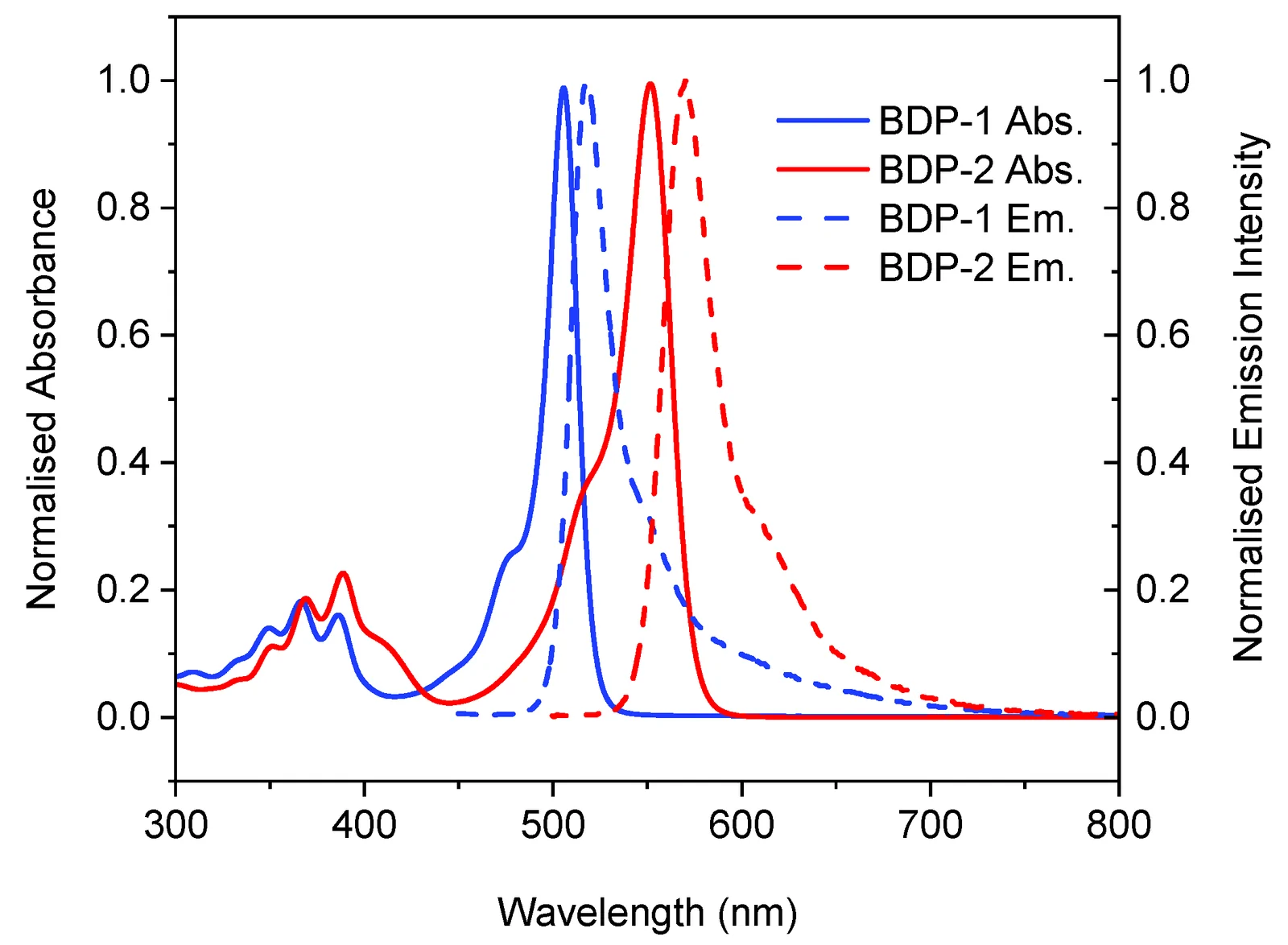
Absorption and emission spectra for **BDP-1** and **BDP-2** in DCM.

The spectra for both BODIPY compounds are displayed in Figure 2. **BDP-1** exhibits the characteristic absorption feature of BODIPYs with a l_max_ at 507 nm corresponding to the S_0_ → S_1_ (π → π*) transition. The lower intensity bands between 320 and 430 nm are assigned to overlapping π → π* transitions on the BODIPY core and the anthracene moiety [30]. The maximum emission is at 552 nm, and a Stokes shift of 344 cm^−1^ was observed in DCM for **BDP-1**. The introduction of the alkyne groups at the 2 and 6 positions extends the conjugation in **BDP-2**, with a 45 nm red-shift in the λ_max_ (552 nm) together with an increased Stokes shift of 633 cm^−1^ in **BDP-2** [31,32,33]. This lower energy absorption band together with the enhanced Stokes’ shift are consistent with theoretical studies (e.g., TD-DFT (Time-Dependent Density Functional Theory)) that predict a bathochromic shift in absorption and emission spectra upon extension of the π-conjugation in BODIPYs [34,35]. These theoretical studies highlight the strong influence of both electron-donating and -withdrawing substituents at the 2,6-positions in tuning the photophysical properties.

An emission lifetime of 4.09 ns was obtained for **BDP-2** (in THF), which is consistent with anthracene BODIPYs in the literature [19]. The emission lifetime of **BDP-1** was 5.69 in THF; however, in MeCN, biexponential signals were obtained with lifetimes of 3.62 ns (23%) and 5.38 ns (77%) (Table 1). **BDP-2** displayed similar characteristics, with a biexponential signal and lifetimes of 2.59 ns (18%) and 5.32 ns (82%), both of which were weak due to the low fluorescence quantum yields. For both compounds, the shorter component is attributed to emission from a CT_s_ state, which is more efficiently populated with increasing solvent polarity. The fluorescence quantum for **BDP-1** is reported in the literature as 0.14, and in this study, a similar value was obtained for **BDP-2** (Φ_Fl_ = 0.19) [36]. This observation agrees with theoretical studies of excited-state dynamics in donor–acceptor BODIPYs that predict enhanced charge-transfer character and solvent-dependent emission in these conjugated BODIPY systems [36]. The emission quantum yields of **BDP-1** and **BDP-2** (Table 1) are consistent with the Reichardt *E_T_* (30) scale of solvent polarities, as opposed to the polarity index, which results in significantly higher emission intensity in THF when compared to their emission in DCM in the case of **BDP-2** [37]. This solvent-dependent behaviour aligns with TD-DFT-supported studies and the influence of solvent polarity on intramolecular charge transfer (ICT) in BODIPY systems, thus reinforcing the proposed mechanism of solvent-stabilised excited states [38].

### 2.2. Time-Resolved Infrared Spectroscopy (TRIR)

**Figure 3:**
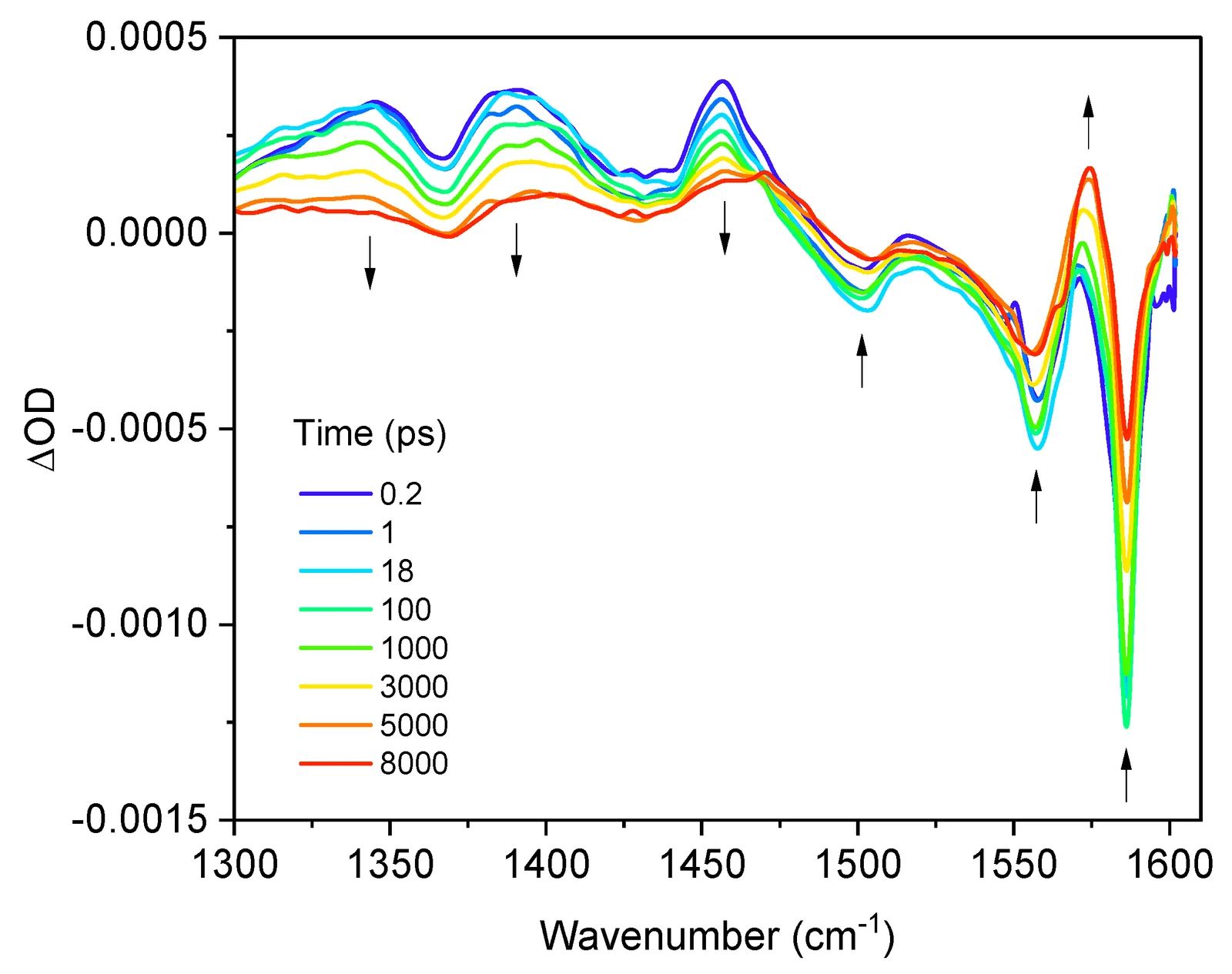
TRIR spectra of **BDP-1** in CDCl_3_, excited at 510 nm (time range, 0.2–8000 ps).

In these experiments the BODIPYs were excited at both 400 and 540 nm, and the infrared spectra were recorded on the femtosecond to picosecond timescale in the range 1300–1600 cm^−1^ (fingerprint region). Following excitation of **BDP-1** at 510 nm in CHCl_3_ the parent features at 1505, 1550, and 1585 cm^−1^ depleted and were accompanied by excited state absorption (ESA) features at 1350, 1390, and 1455 cm^−1^ (Figure 3).

The ESA bands decay significantly over 8000 ps, however, on the timescale. These spectral infrared bands are assigned to vibrational modes centred on the BODIPY core and the phenyl moieties and are attributed to π → π* transitions [40]. Global analysis of the spectra yielded two lifetimes, which are tentatively assigned to intersystem crossing and population of the singlet state. The shorter lifetime of 18.93 ps is likely due to intersystem crossing due to SOCT-ISC following photoinduced electron transfer (PeT) from the anthracenyl moiety to the BODIPY core in a π_An_ → π_BDP_ transition, leading to population of the hole in the BODIPY HOMO following excitation. PeT has previously been reported by Filatov et al. for **BDP-1** (2.4 ps), and in this study gives rise to the vibrational bands in the TRIR spectra [41]. The longer lifetime of 7.3 ns is assigned to the S_1_ state. As features persist in the TRIR spectra beyond the experimental window (8 ns), these bands are attributed to population of one or more triplet states, which is known to occur in this BODIPY [39].

**Figure 4:**
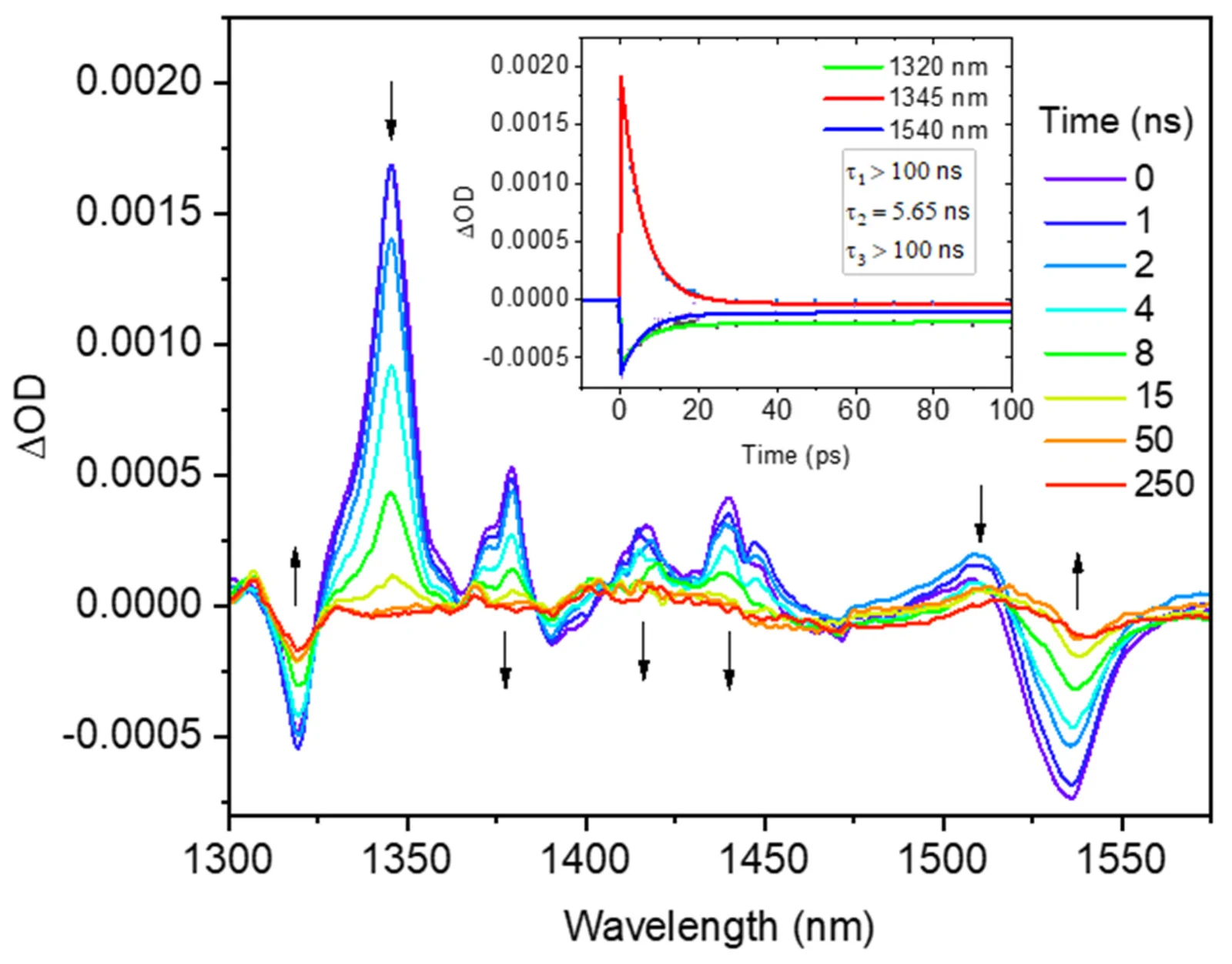
TRIR of **BDP-2** in CDCl_3_, excited at 540 nm (time range, 0–250 ns).

Following excitation of **BDP-2** at 540 nm (Figure 4), GSBs occur at 1320 and 1540 cm^−1^ with the concomitant formation of ESA bands at 1306, 1345, 1380, 1417, 1439, and 1510 cm^−1^. Global Analysis of the spectra yielded a lifetime of 14.15 ps, which is assigned to ISC, as observed for **BDP-1**, and is attributed to SOCT-ISC following PeT from the anthracene to the BODIPY core. The longer lifetime of 6.57 ns is assigned to the population of the S_1_ state. While the spectral features differ between **BDP-1** and **BDP-2**, similar kinetics were obtained. Also, for **BDP-2**, ESA features persist beyond 9 ns and again are assigned to an excited triplet species.

**Figure 5:**
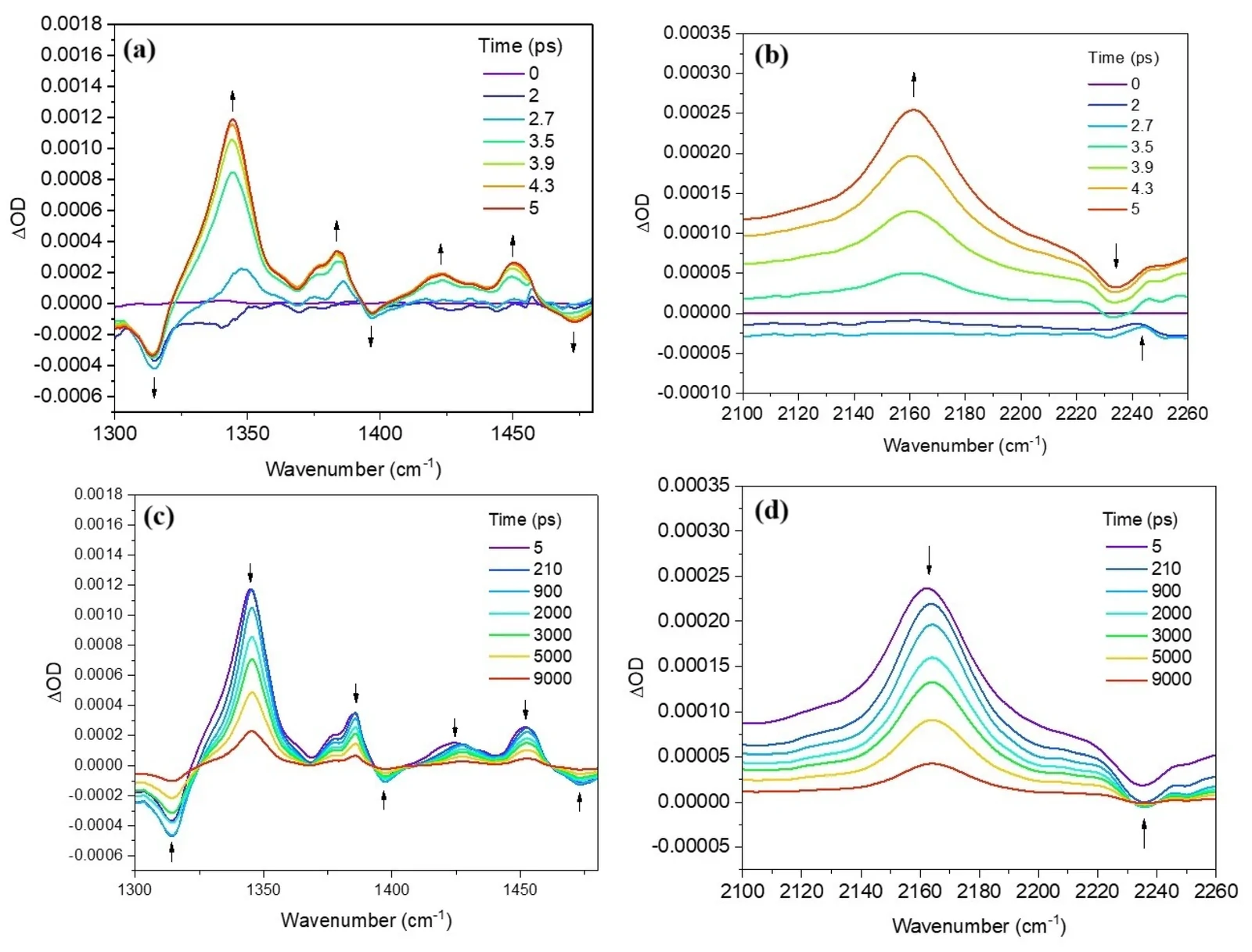
TRIR spectra following excitation of **BDP-2** in CDCl_3_ at 400 nm (0–5 ps): (**a**) In the fingerprint region, (**b**) in the alkynyl region, and (**c**) the fingerprint region from 5 to 9000 ps. (**d**) In the alkynyl region from 5 to 9000 ps.

The TRIR spectra for **BDP-2** displayed in Figure 5 display differences following excitation at 400 nm when compared to the results following excitation at 540 nm. Excitation at 540 nm lies in the region of the S_1_ band, while absorption of a 400 nm photon may populate excited states on the BODIPY core or the anthracene moiety. Each pathway may subsequently result in the population of triplet states via charge transfer intermediates. Following excitation, GSBs were observed at 1314, 1397, and 1475 cm^−1^, and over the subsequent 5 ps, new bands grew in at 1346, 1385, 1423, and 1452 cm^−1^ together with an ESA band at 2250 cm^−1^ in the triple bond region.

The notable change in absorbance of the bands over 5 ps, especially in terms of the growth of the band at 2163 cm^−1^ highlights a significant change in dipole moment across the photosensitiser. The ESA at 2163 cm^−1^ has shifted to a lower wavenumber with respect to its corresponding GSB at 2250 cm^−1^, indicating a loss of electron density in the alkynyl moieties at the 2 and 6 positions. This, in combination with the enhanced amplitude of the ESA, is indicative of a charge transfer from the alkynyl bonds to the BODIPY, which is also evidenced by the large increase in the intensity of the ESA bands in the fingerprint region, particularly at 1345 cm^−1^ [42]. The enhanced alkynyl ESA band appears at a higher energy than a similar system reported by Cullen et al., who observed the formation of this band at 2073 cm^−1^ in the BODIPY-diethynylbenzene copolymers, which is 90 cm^−1^ lower than for **BDP-2** [43]. This can be attributed to the lack of the phenyl component conjugated to the ethynyl bond in **BDP-2**. The decrease in π-conjugation in BDP-2 when compared to Cullen’s copolymer allows less resonance and delocalisation of π-electrons in the triple bond, resulting in vibrational modes at a higher energy [43].

### 2.3. Nanosecond Transient Absorption Spectroscopy (TAS)

Nanosecond-transient absorption spectroscopy was conducted to determine the triplet lifetimes for **BDP-1** and **BDP-2** in different solvents. The samples were prepared with an absorption value of ~0.3 at the excitation wavelength (355 or 532 nm) and deaerated using Schlenk technique. This is essential, as any oxygen present can quench the excited triplet states of the photosensitiser. The photosensitisers were irradiated at 355 nm (except **BDP-2** in DCM to avoid radical formation in the chlorinated solvent), and spectral changes in the visible region were monitored.

**Figure 6:**
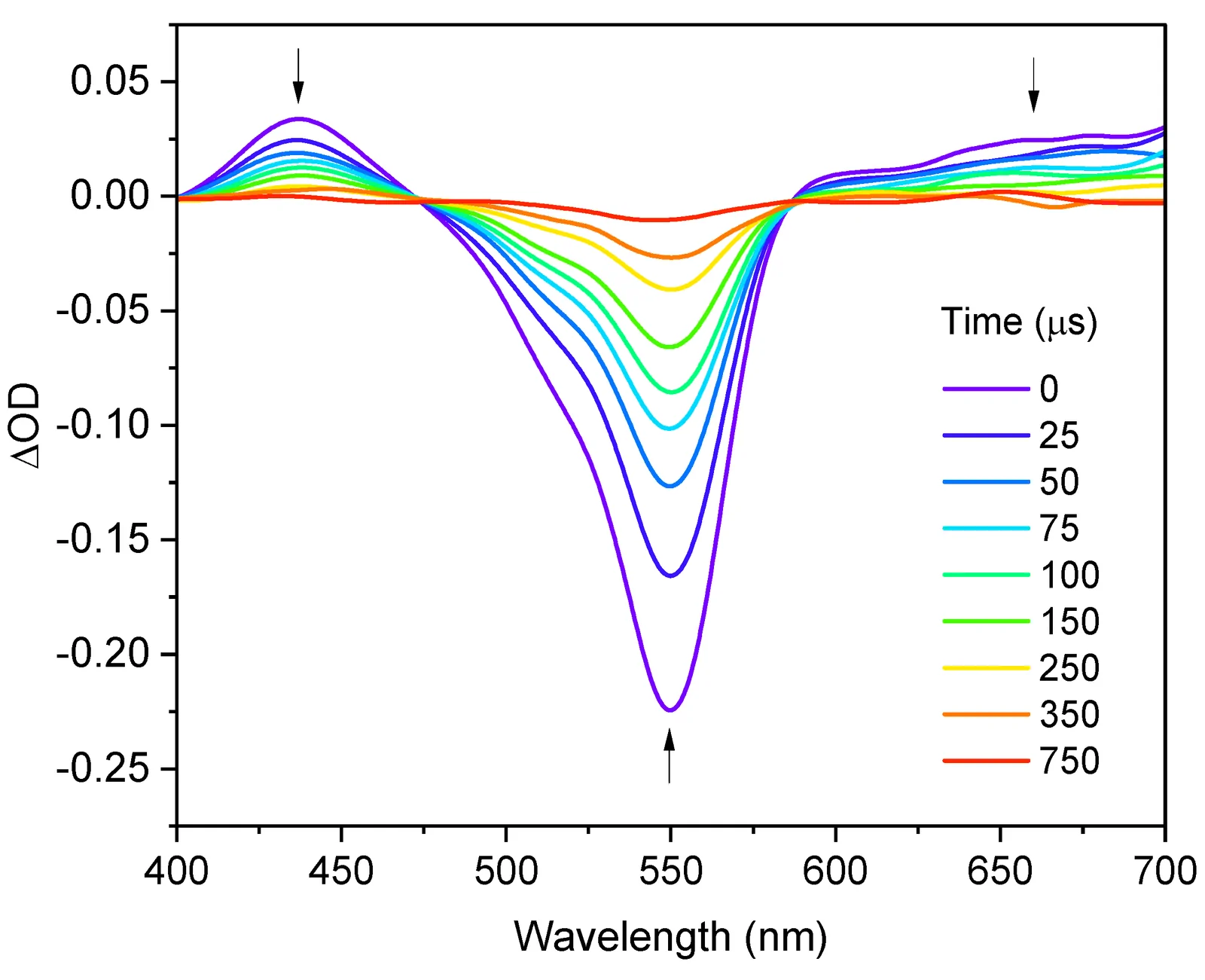
Nanosecond transient absorption (ns-TAS) of **BDP-2** in DCM, following excitation at 532 nm (0–750 µs).

An ns-TAS spectrum is shown in Figure 6 following excitation of **BDP-2** at 532 nm in DCM. A ground-state bleach (GSB) occurs in the region 480–580 nm, corresponding to a depopulation of the ground state of the photosensitiser, accompanied by an excited state absorption (ESA) feature between 400 and 470 nm. As the ESA feature decays on the microsecond time scale, the GSB also recovers, indicating a population of S_0_. The time at which this decay occurs is indicative of relaxation from a triplet state to the ground state [43]. **BDP-1** and **BDP-2** exhibited two lifetimes in the microsecond time range. The triplet lifetimes for **BDP-2** (τ = 31 and 138 µs, in MeCN) were generally longer-lived than those of **BDP-1** (τ = 18 and 105 µs, in MeCN) (see Table 2), although all were significantly longer than those reported for heavy-atom-containing BODIPYs [43,44]. The presence of two long-lived components in the ns-TAS results supports the assumption that the population of the T_1_ triplet state is preceded by charge transfer intermediates. In general, the shorter-lived component contributes to ~30% of the decay curve, and the longer-lived component accounts for 70% of the signal. SOCT-ISC begins to emerge again as a viable pathway for these photosensitisers, with the longer component assigned to the T_1_ state and the shorter component likely a CT_T_ state. As the population of the CT_T_ state must be preceded by the population of the CT_S_ state, it is feasible that the population of T_1_ occurs directly from CT_S_ and CT_T_ after charge recombination, with a change in orbital-angular momentum accompanying the charge recombination in the former. This has been extensively reported in work conducted by Filatov and Wang and coworkers and alluded to by TR-EPR [18,39].

### 2.4. Quantification of Singlet Oxygen

Two well-known methods for quantification of singlet oxygen quantum yields in photosensitisers are by direct measurement of the phosphorescence of singlet oxygen at 1270 nm and by indirect measurement by using singlet oxygen scavengers such as 1,3-diphenylisobenzofuran and monitoring the decay of this scavenger over time under irradiation of the compound. It is important to note that this method is only suitable for irradiation at wavelengths where the scavenger does not absorb, as it is very sensitive to light. It should be noted that anthracene itself is also a singlet oxygen scavenger, and the addition of oxygen to the anthracene rings quenches its triplet excited states [41]. As a result, we found the indirect method to be more suitable as a means of detection, as on its own the anthracene would be the only scavenger available for the direct method; however, when the DPBF scavenger is present, it would be in competition for the singlet oxygen and yield a more accurate estimation of the singlet oxygen quantum yield.

In these experiments **I_2_-BDP** was used as a standard, as it has a reported singlet oxygen quantum yield of 0.87 [39,45], its structure is shown in Figure S33. The singlet oxygen quantum yields of **BDP-2** were consistent with those of **BDP-1,** which have previously been reported in the literature [15,17,18]. The lowest yields were seen in THF; however, this was accompanied by the highest fluorescence quantum yield. Both BODIPYs displayed high singlet oxygen quantum yields in DCM and MeCN; however, the fluorescence quantum yields in MeCN were heavily diminished (0.01), whereas they retained a significant amount of fluorescence in DCM (0.10–0.20). These results show overall high singlet oxygen quantum yields upon irradiation in the visible region, making both **BDP-1** and **BDP-2** suitable candidates for biological testing (Figures S21–S31). A control experiment was conducted by irradiating a solution of DPBF in the absence of the photosensitisers under identical conditions. No significant change in the absorption at 414 nm was observed (Figure S32), confirming that DPBF degradation in the main experiments arises from singlet oxygen generated by the photosensitisers.

### 2.5. Biological Studies

#### 2.5.1. BSA-Binding Studies

BODIPY dyes, known for their high fluorescence, photostability, and environmental sensitivity, are widely used as molecular probes. When conjugated with BSA, a protein with multiple well-defined binding sites, the fluorescence properties of BODIPY can change, providing insights into protein-ligand interactions, structural dynamics, and conformational changes. Understanding this interaction is valuable for applications in drug delivery and biosensor development [46,47].

This study used fluorescence spectroscopy to investigate the binding between **BDP-2** and BSA. This technique enables evaluation of binding affinity, protein conformational changes, and environmental effects on the fluorophore. Changes in fluorescence intensity and quenching reveal binding characteristics, site interactions, and structural modifications in BSA upon BDP-2 binding, offering valuable data for biochemical research [48,49].

**Figure 7:**
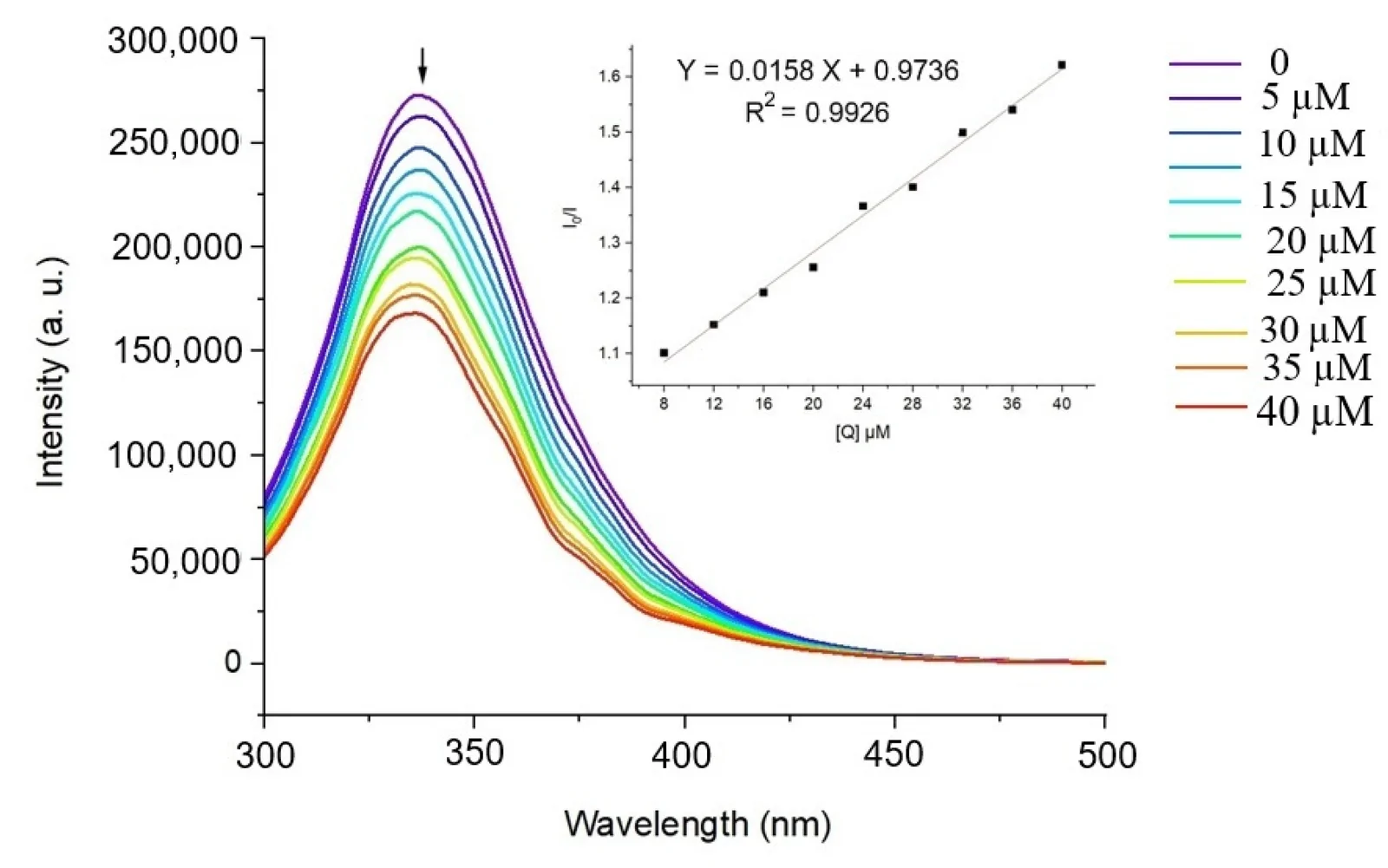
Fluorescence quenching curves of BSA (50 µM) in PBS solution in the presence of different amounts (0–40 µM) of **BDP-2**. Insert: Stern–Volmer plot of the fluorescence titrations.

BSAs intrinsic fluorescence mainly arises from its aromatic residues: tryptophan, tyrosine, and phenylalanine. Tryptophan, particularly Trp-213 located in a hydrophobic pocket, is the dominant contributor due to its high quantum yield and environmental sensitivity, with an emission peak around 330–350 nm. Tyrosine (303 nm) and phenylalanine (282 nm) contribute less due to lower quantum yields and quenching effects [50,51]. These properties make BSA fluorescence a sensitive indicator of conformational changes and molecular interactions [49,52]. The fluorescence emission spectra of BSA in the presence of varying concentrations of **BDP-2** are presented in Figure 7. A decrease in fluorescence intensity at 338 nm was observed, indicative of quenching effects caused by **BDP-2** binding to BSA. The degree of quenching was quantitatively analysed using the Stern–Volmer equation, from which the Stern–Volmer constants K_SV_ (*I*_0_/*I*= 1+ *K*_SV_ [Q]) were determined. The K_SV_ value was 1.58 × 10^4^ M^−1^ for **BDP-2** (insert Figure 7). The quenching constant (K_q_), where τ_0_ is the lifetime of the fluorophore (BSA) in the absence of the quencher (10 ns) [53], yielded K_q_ = 1.58 × 10^12^ M^−1^ S^−1^ for **BDP-2**. The fluorescence quenching constant (K_q_) for **BDP-2**, which exceeds 10^12^ M^−1^ s^−1^, suggests the presence of a statistical interaction mechanism at play [54].

For a static quenching interaction, the fluorescence intensity data can also be used to determine the apparent binding constant (*K*_b_) and the number of BSA binding sites (*n*) for the complex using the following equation [54]:

log ((*I*_0_ − *I*)/*I*) = log *K*_b_ + *n* log [Q]

From the plot of log ((*I*_0_ − *I*)/*I*) versus log [Q] (Figure S34), the number of binding sites (*n*) and the binding constant (*K*_b_) values were calculated.

For **BDP-2**, a linear relationship is observed with binding constants K_b_ of 9.6 × 10^4^ M^−1^ S^−1^. In both compounds, the value of *n* was determined to be 1.17, which is close to 1. This indicates only one binding site for **BDP-2** on the BSA molecule. When compared to literature data for similar compounds, this value is higher than that of a water-soluble meso-thienyl BODIPY derivative (K_b_ = 4.15 × 10^4^ M^−1^) [55] and a BODIPY-benzimidazole conjugate (K_b_ = 1.38 × 10^4^ M^−1^) [56], suggesting that **BDP-2** binds more effectively to BSA. Moreover, naturally derived compounds like curcumin and its piperazine derivative displayed much lower binding constants (K_b_ = 1.0 × 10^4^ M^−1^ and 9.1 × 10^3^ M^−1^, respectively), further emphasising the superior binding strength of **BDP-2** [57].

The quenching constant (K_q_) for **BDP-2** was calculated as 1.58 × 10^12^ M^−1^ S^−1^, which is notably higher than typical quenching constants observed in similar systems, generally ranging from 10^9^ to 10^11^ M^−1^ S^−1^. This elevated quenching constant suggests that **BDP-2** interacts with BSA through a highly efficient quenching mechanism, likely involving dynamic or statistical processes. The binding interaction between **BDP-2** and BSA holds potential for various biomedical applications. Conjugation with BSA can enhance the solubility, biocompatibility, and targeted delivery of BODIPY-based probes. Such complexes are particularly useful in controlled drug delivery, diagnostic imaging, and as biosensors, where changes in fluorescence can signal molecular binding events or environmental shifts. Therefore, although **BDP-2** exhibits superior binding affinity and quenching efficiency compared to other BODIPY derivatives, further investigation into its biocompatibility is warranted before considering its use in biological systems.

#### 2.5.2. Biocompatibility Investigation

**Figure 8:**
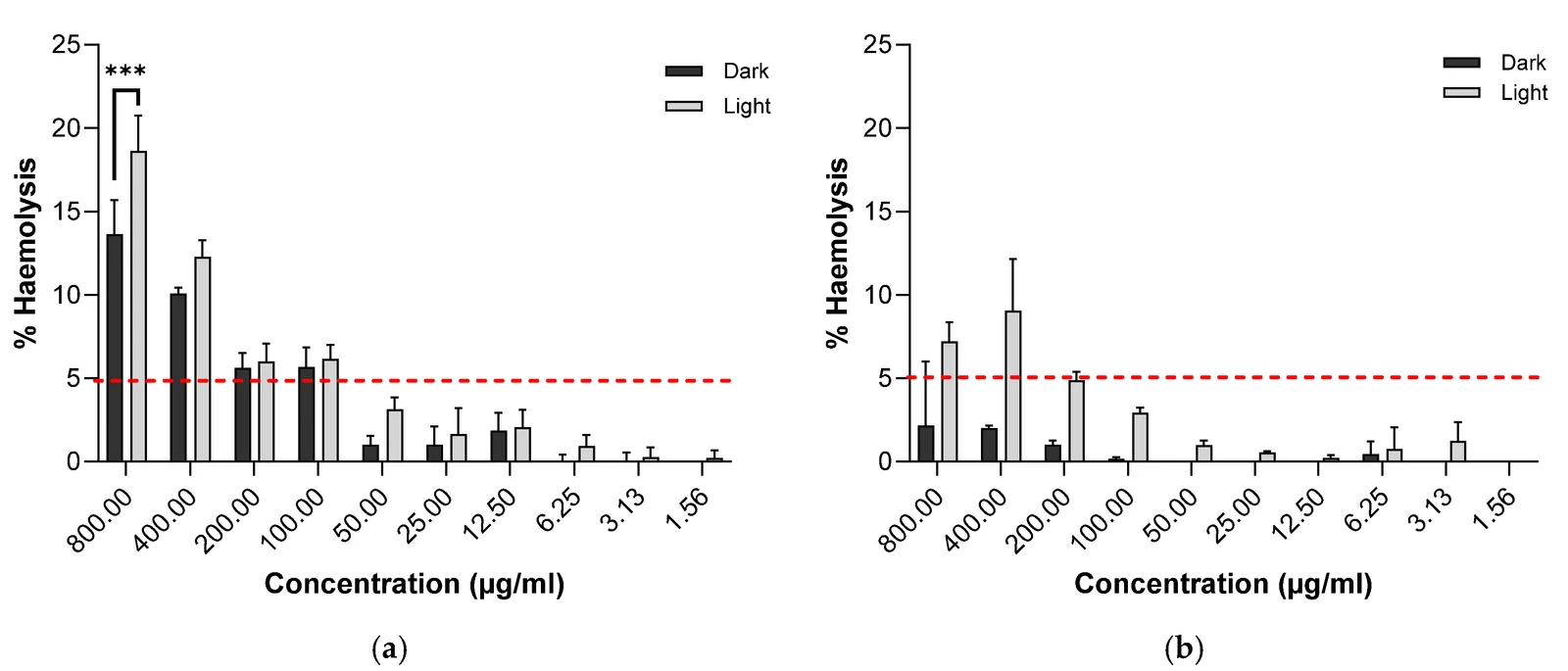
In vitro haemolysis studies of human erythrocytes for (**a**) BDP-1 and (**b**) BDP-2 at varying concentrations under dark conditions and when irradiated at 370 nm. Values shown are the mean ± SEM for triplicate assays. Statistically significant differences between the light and dark series are shown: *** *p* ≤ 0.0001. The red dotted line indicates 5% haemolysis.

The haemolysis of human erythrocytes in vitro is used as a measure of biocompatibility, with a threshold of 5% haemolysis or less applied. Under irradiation conditions (370 nm, 1 h), **BDP-1** displayed greater than 5% haemolysis at concentrations of 50 μg·mL^−1^. However, improved biocompatibility was found for **BDP-2** under the same conditions, with haemolysis remaining below the 5% threshold up to 200 μg·mL^−1^ (Figure 8).

#### 2.5.3. Antibacterial Studies

**Figure 9:**
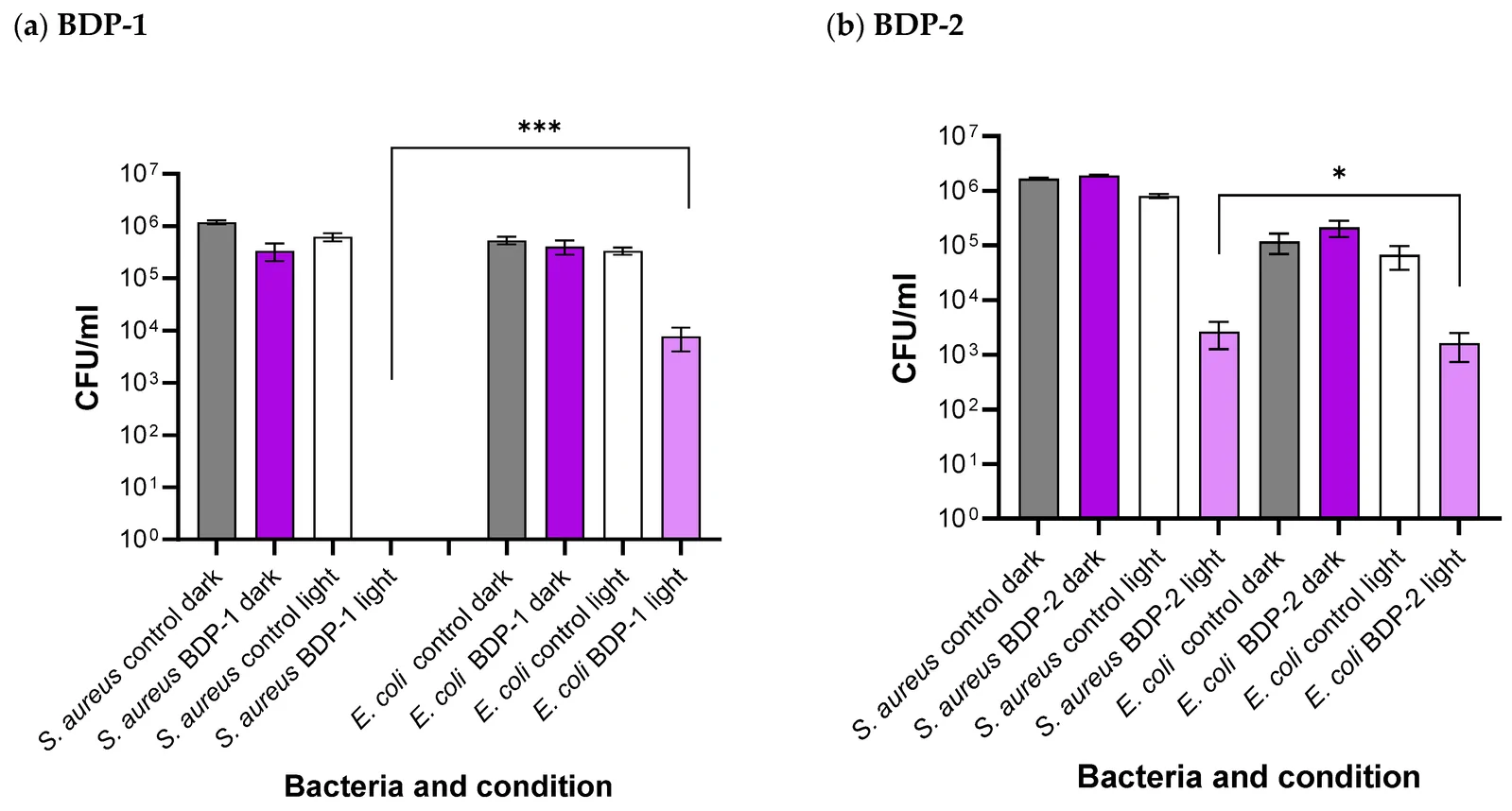
Photodynamic bactericidal activity of **BDP-1** (**a**) and **BDP-2** (**b**) against two bacterial species. Assays containing 10% DMSO (controls) or compounds (100 μg·mL^−1^) and approximately 10^6^ CFU·mL^−1^ bacteria were incubated in the dark or irradiated for 60 min at 370 nm. Values shown are mean ± SEM for three separate assays performed in triplicate. The Y axis is logarithmic scale. Statistically significant differences between the reduction in CFU/mL for light-treated series (control minus BDP-treated) against *E. coli* vs. *S. aureus* are shown: * *p* ≤ 0.05, *** *p* ≤ 0.0001. For BDP1 with light treatment, bars representing zero CFU/mL are not visible.

**Figure 10:**
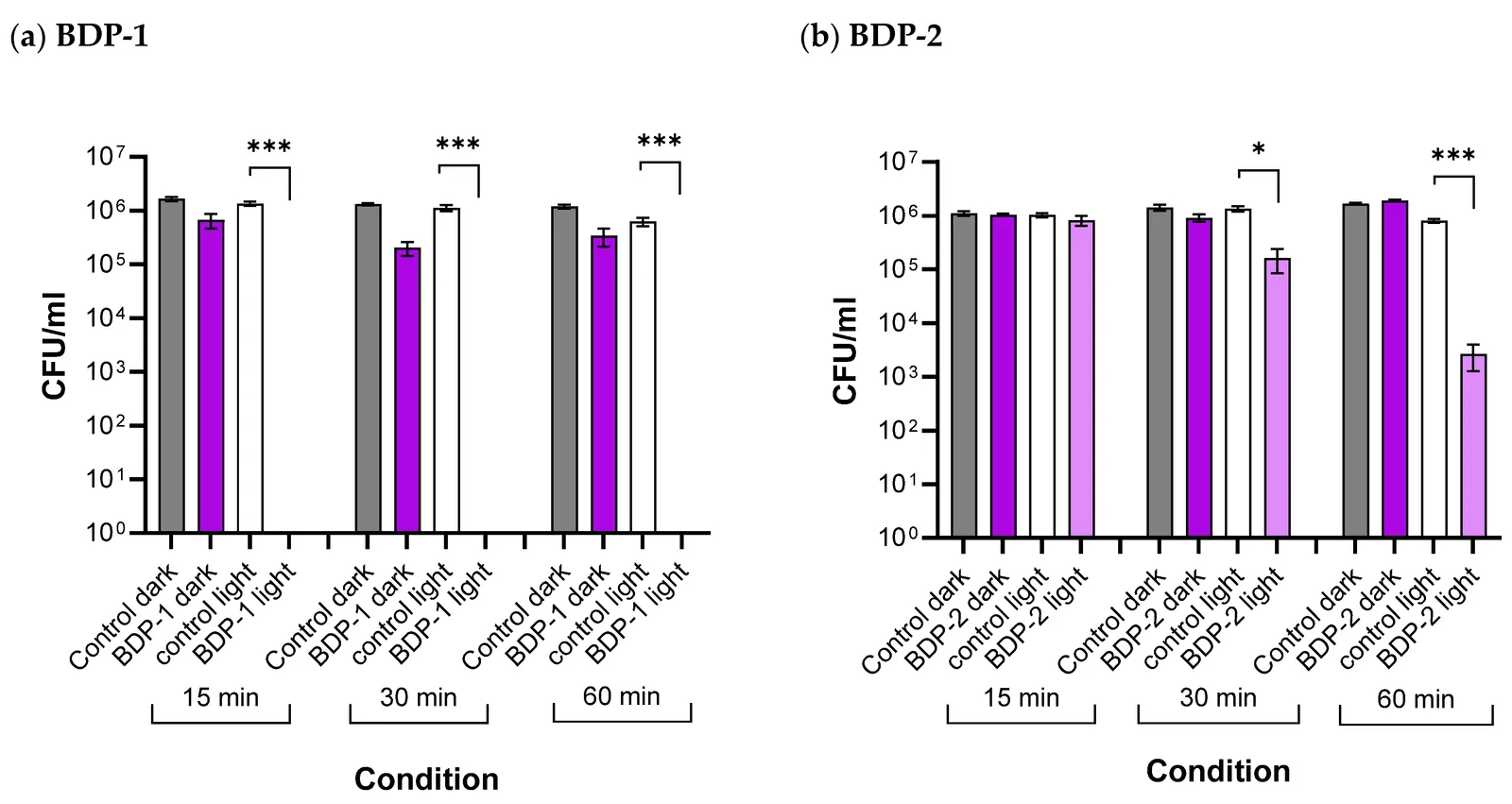
Photodynamic bactericidal activity of (**a**) **BDP-1** and (**b**) **BDP-2** against *S. aureus* (ATCC 25923). Assays containing 10% DMSO (controls) or compounds (100 μg·mL^−1^) and approximately 10^6^ CFU·mL^−1^ bacteria were incubated in the dark or irradiated at 370 nm for 15, 30, and 60 min. Values shown are mean ± SEM for three separate assays performed in triplicate. The Y axis is a logarithmic scale. Statistically significant differences between the reduction in CFU/mL for the light-treated series (control minus BDP-treated) are shown: *** *p* ≤ 0.001, * *p* ≤ 0.05. For BDP1 with light treatment, bars representing zero CFU/mL are not visible.

**Figure 11:**
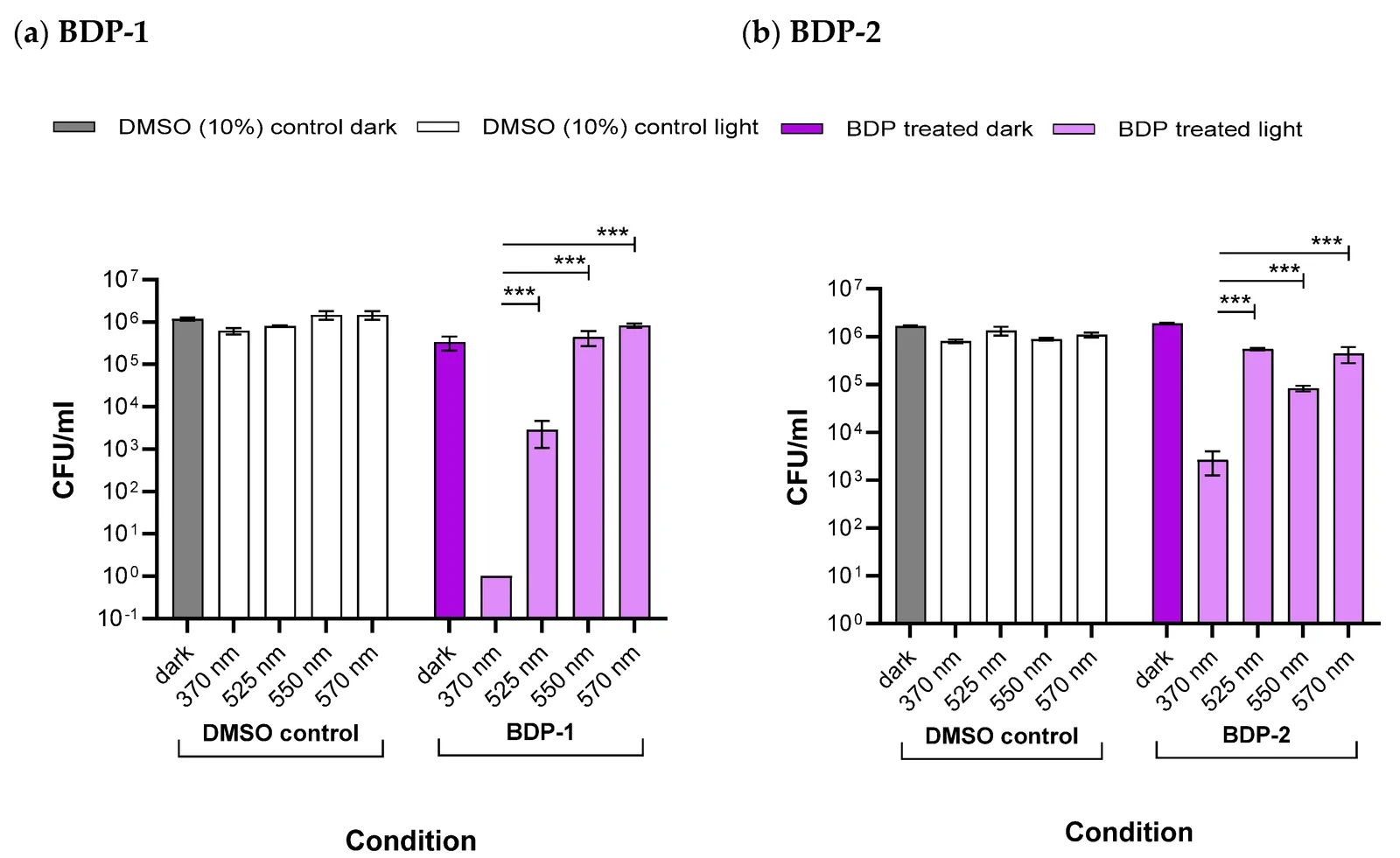
Effect of irradiation wavelength on antimicrobial activity of (**a**) **BDP-1** and (**b**) **BDP-2**. Bacterial killing activity at 100 μg/mL is shown against *S. aureus* (ATCC 25923). Assays were incubated in the dark or irradiated for 60 min at 370, 525, 550, and 570 nm. Values shown are mean ± SEM for three separate assays performed in triplicate. Statistically significant differences between the reduction in CFU/mL for light-treated series (control minus BDP-treated) are shown: *** *p* ≤ 0.001.

Under irradiation at 370 nm for 1 h, the highest level of killing was seen in *S. aureus,* a Gram-positive bacterial species, where a 6 log reduction in CFU/mL was found for **BDP-1** compared to only 2.68 log for **BDP-2**. Both **BDP-1** and **BDP-2** had lower activity against Gram-negative *E. coli*, with killing activity of 1.74 and 1.30 log, respectively (Figure 9). An investigation of the time course of *S. aureus* killing showed differences between **BDP-1** and **BDP-2**. For **BDP-1**, 6 log killing was achieved, even at the lowest period of irradiation (15 min), but for **BDP-2**, this pattern was not observed; rather, time dependence was apparent, with at least 1 h of irradiation required to reach 2.7 log (Figure 10). The differences in bactericidal activity for **BDP-1** and **BDP-2** could be due to the structural differences and interactions with the bacterial cells and also the light dose from the LED lamps used (Table 3, Figure 11). Control experiments with irradiation at 370 nm alone for 60 min had a negligible effect on bacterial viability, and therefore, killing required the combination of 370 nm and BDPs.

## 3. Materials and Methods

All chemicals and solvents were supplied by Aldrich Chemicals Co. (Wicklow, Ireland) and anhydrous solvents containing Sure/Seal were used under the flow of nitrogen. The NMR spectra were recorded on a Bruker 600 MHz spectrometer (Tospin 2.1, Bruker BioSpin srl, Milan, Italy). Mass spectroscopy was measured on a maXis HD (Bruker, Bremen, Germany) with an Ion trap detector interfaced to an Agilent 1100 series HPLC system (Agilent, Santa Clara, CA, USA). The BODIPYs were synthesised by methods previously reported in the literature. **BDP-1** was synthesised using standard procedures reported in the literature [39]. **BDP-1a** was synthesised by iodination of **BDP-1** under similar conditions reported by Cullen et al. with minor modifications [43]. **BDP-2** was synthesised by Sonogashira coupling of **BDP-1a** with phenylacetylene under similar conditions to those reported by Zhang et al. [58,59]. Absorption, excitation, and emission spectra were recorded using a Horiba Fluorimeter (Kyoto, Japan). Samples were aerated and prepared to an optical density of <0.2 at the excitation wavelength in quartz cuvettes. Emission lifetimes and fluorescence quantum yields for the BODIPY samples were recorded on the Time-correlated Single Photon Counter (TCSPC) from Edinburgh Instruments. Samples were aerated and prepared to an optical density of <0.2 at the excitation wavelength in quartz cuvettes. Nanosecond transient absorption spectroscopy spectra were recorded on the LP980 transient absorption spectrometer from Edinburgh Instruments (Edinburgh, Scotland). Samples were prepared to an optical density of < 0.4 at the excitation wavelength in the absence of oxygen, by performing three freeze-pump-thaw cycles under the Schlenk technique. Singlet oxygen quantum yields were recorded using a Shimadzu Spectrometer (Shimadzu, Kyoto, Japan) and 1,3-Diphenylisobenzofuran (DPBF) from Merck (Arklow, Ireland) as the scavenger. A lamp with an irradiation wavelength of 528 nm was used.

### 3.1. Protein Binding Studies

The binding of **BDP-2** to BSA was investigated using fluorescence spectroscopy recorded at a fixed excitation wavelength (280 nm) corresponding to BSA while monitoring the emission at 335 nm at 298 K. The stock solution of BSA was prepared in 50 mM phosphate buffer (pH = 7.2) and stored in the dark at 4 °C for further use. Concentrated stock solutions of each test compound were prepared by dissolving the compound in phosphate buffer and subsequently diluted with phosphate buffer to the required concentrations. Aliquots of 0–40 µM test solutions were added to BSA (50 µM). A 3 mL solution containing BSA (50 µM) was titrated by successive additions of 0–40 µM test solutions (**BDP-2**) for fluorescence measurements.

### 3.2. Singlet Oxygen Studies

Singlet oxygen quantum yields were determined using 1,3-Diphenylisobenzofuran (DPBF) as a scavenger. DPBF reacts with singlet oxygen, resulting in a decrease in its absorption maxima at 414 nm. The rate of this decrease can be correlated to the amount of ^1^O_2_ produced by the PS using the formula [41]:

$${ɸ}_{\Delta }={ɸ}_{std}\left(\frac{m_{BDP}}{m_{std}}\right)\left(\frac{\alpha_{std}}{\alpha_{BDP}}\right)$$

where ɸ_Δ_ is the singlet oxygen quantum yield of the analyte, ɸ_std_ is the singlet oxygen quantum yield of the standard (**I_2_-BDP**, Figure S32) [31,41], *m* is the normalised slope of the curve monitoring changes in absorption at 414 nm over time (s), and α = 1–10^−A^, where A is the average absorption of the sample or standard at the irradiation wavelength (528 nm). Samples were irradiated using a 528 nm LED light source with a measured power output of 0.152 mW/cm^2^. The samples were placed in a 1 cm path length quartz cuvette and irradiated for up to 180 s, resulting in a total light dose of 28 mJ/cm^2^. The distance from the LED source to the sample surface was maintained at 30 cm. Absorbance changes were recorded at fixed intervals using a UV–vis spectrophotometer.

### 3.3. Antimicrobial Studies

Stock solutions of the BODIPYs were prepared freshly on the day of testing by dissolving in 100% (*v*/*v*) Dimethyl sulfoxide (DMSO) at 1 mg·mL^−1^. All stocks were stored in the dark until required. Bacterial strains *Staphylococcus aureus* (ATCC25922) and *Escherichia coli* (ATCC25922) were purchased from the Health Protection Agency, UK. Pure colonies were isolated by subculturing on Mueller–Hinton agar and overnight aerobic growth at 37 °C in a static incubator. A bacterial suspension was prepared from single colonies with phosphate buffered saline (PBS) to the density of a 0.5 McFarland standard using a pre-calibrated turbidometer (Densichek TM, Biomerieux, Marcy-L’Etoile, France). The suspension was further diluted ^1^/_100_ in PBS. Assays were prepared in the wells of 96-well plates by adding 20 μL of stock BODIPY solution to triplicate wells and 180 μL of diluted bacterial suspension, giving a final assay concentration of 100 μg·mL^−1^ and approximately 10^6^ colony-forming units/mL (CFU·mL^−1^) of bacteria. Two control series were prepared in which BODIPYs were replaced by either diluent (PBS) or 10% DMSO. Duplicate plates were prepared in this way: One was incubated at room temperature in the dark for various times (15–60 min), and the other was irradiated with light for the same time period. Therefore, each experiment included bacteria in the dark with no BDPs, irradiated bacteria with no BDPs, bacteria in the dark with BDPs, and irradiated bacteria with BDPs. The lamps used were suspended above the plate at a distance of 30 cm. After irradiation, ^1^/_10_ serial dilutions were prepared from treated and control wells under light and dark conditions in PBS from 10^−1^ to 10^−4^. Aliquots (20 μL) from these dilutions were applied in triplicate to four quadrants of Mueller–Hinton agar plates. The plates were incubated overnight at 37 °C in a static incubator. CFU·mL^−1^ was determined by counting resulting colonies at each dilution in treated and control plates.

### 3.4. Synthesis

#### 3.4.1. Synthesis of 1-(tert-Butyl) 4-(Prop-2-yn-1-yl) Piperazine-1,4-Dicarboxylate (Boc-PP)

Approximately 500 mg of 1-Boc-piperazine was added to anhydrous dichloromethane (DCM, 30 mL) and TEA (1 mL). The mixture was purged under N_2_ for 15 min and allowed to react overnight under an N_2_ atmosphere with stirring. The mixture was initially placed in an ice bath when adding propargyl chloroformate (395 µL, 1.5 eq.) to control the reaction rate. The crude mixture was dried under reduced pressure and redissolved in DCM (40 mL) and washed with saturated Na_2_CO_3_ solution (30 mL (×3)). The organic phase was then dried over MgSO_4_ and filtered by gravity filtration and then dried under reduced pressure. No further purification was required. This afforded a white solid (94%). ^1^H NMR (600 MHz): 4.64 (s, 2H, ^4^*J* = 2.3 Hz), 3.38 (m, 8H), 2.45 (t, 1H, ^4^*J* = 2.3 Hz), 1.40 (s, 9H).

#### 3.4.2. Synthesis of BDP-2

The product was synthesised through Sonogashira. Coupling with palladium as co-catalysts, as per previously reported methods with minor modifications [43]. **BDP-1a** (200 mg, 0.32 mmol) and Boc-piperazine-propargyl (BPP) (188 mg, 0.70 mmol) were added to a dry Schlenk tube with a magnetic stir bar. In addition to this, tetrakis(triphenylphosphine)palladium(0) Pd(PPh_3_)_4_ (37 mg, 10 mol%) and copper(I) iodide (CuI) (7 mg, 10 mol%) were added to the Schlenk tube. Tetrahydrofuran (THF, 10 mL) and triethylamine (TEA, 10 mL) were added to the Schlenk tube. The mixture was then freeze-pump-thawed under high vacuum strength and nitrogen flow using the Schlenk technique. It was then brought to a temperature of 80 °C while stirring for 24 h. The crude mixture was dried under reduced pressure and redissolved in DCM (40 mL) and washed with saturated Na_2_CO_3_ solution (30 mL (×3)). The organic phase was then dried over MgSO_4_ and filtered by gravity filtration. The product was then purified by silica column chromatography with a gradient mobile phase of DCM and ethyl acetate (starting at 95:5) to afford a purple solid (17%). ^1^H NMR (600 MHz, CDCl_3_): *ẟ* = 8.60 (s, 1H), 8.04 (d, 2H, ^3^*J* = 8.6 Hz), 7.76 (d, 2H, ^3^*J* = 8.6 Hz), 7.48 (t, 2H, ^3^*J* = 7.5 Hz), 7.40 (t, 2H, ^3^*J* = 7.7 Hz), 4.82 (s, 4H), 3.36 (m, 16H), 2.68 (s, 6H), 1.43 (s, 18H), 0.70 (s, 6H). ^13^C NMR (CDCl_3_): δ = 159.23, 154.69, 154.52, 145.21, 140.83, 132.09, 131.44, 129.60, 129.00, 128.68, 127.45, 127.32, 126.02, 124.78, 115.35, 90.80, 80.32, 78.53, 54.13, 43.81, 29.81, 28.49, 13.87, 12.44. ESI-MS = 957.4471 *m*/*z*, [M + H]^+^.

## 4. Conclusions

In this study, a piperazine-containing side chain integrated into the BODIPY structure (**BDP-2**) was synthesised and assessed for antibacterial activity. The high-yield iodination of **BDP-1** to form **BDP-1a** and the subsequent Sonogashira coupling to generate **BDP-2** highlight the synthetic adaptability of the BODIPY framework. A significant redshift and increased Stokes shift were observed for **BDP-2,** which is attributed to structural modifications. The BODIPYs presented in this work exhibit long-lived excited states and charge transfer characteristics, providing strong evidence that SOCT-ISC is the predominant pathway leading to population of the triplet state. Incorporation of the piperazine-containing side chain into the BODIPY structure not only resulted in a significant red-shift in absorption but also longer-lived triplet states compared to its parent anthracene BODIPY. Time-resolved techniques, such as TRIR and TAS, confirmed the efficient formation of these triplet states while also highlighting the influence of solvent polarity and structural modifications on the photophysical behavior. These findings offer valuable insights into how these compounds can be optimised for specific applications, such as antimicrobial photodynamic therapy, by fine-tuning their emission lifetimes, quantum yields, and triplet-state formation. This study lays the groundwork for the development of BODIPY-based photosensitisers with enhanced efficacy for photophysical and photobiological applications.

Biological studies demonstrated strong binding interactions between **BDP-2** and BSA, indicating its potential for drug delivery systems. Furthermore, both compounds exhibited high singlet oxygen yields in polar solvents, validating their utility in photodynamic therapy and other biological applications.

The antibacterial assays further highlighted the potential of **BDP-1** and **BDP-2** as photosensitisers, particularly against gram-positive *S. aureus*, where potent bactericidal activity was observed. This *S. aureus*-specific activity informs the potential clinical applications of these compounds. *S. aureus*-specific eradication capability would favour their development for *S. aureus* aPDT decolonisation strategies, for example, prior to surgery. Currently, decolonisation (for methicillin-resistant *S. aureus*—MRSA) relies on nasal mupirocin application or chlorhexidine bathing. Furthermore, targeted treatments are central to antimicrobial stewardship programs to avoid the development of AMR associated with broader-spectrum agents, and in this regard, particularly for skin and soft tissue infection, where *S. aureus* predominates as the infecting pathogen, aPDT using specific BDPs to target *S. aureus* would be impactful. However, under the experimental condition used in our studies, the efficacy of **BDP-1** and **BDP-2** was wavelength-dependent, with superior antibacterial performance at 370 nm compared to 525 nm. This could be attributed to several factors, including population of the triplet state and light dosage.

In conclusion, **BDP-1** and **BDP-2** are promising candidates for antibacterial applications where *S. aureus* targeted eradication is required clinically, and future work will focus on exploring structure-property relationships and more extensive antibacterial studies under varying conditions.

## Figures and Tables

**Table 1:** Photophysical results for **BDP-1** and **BDP-2** in various solvents.

	Solvent	abs (nm)	em (nm)	Δ$\bar{\upsilon }$ (cm^−1^)	ɸ_Fl_	τ_1_ (ns)	τ_2_ (µs)	ɸ_Δ_
**BDP-1**	DCM	507	516	344	0.14 ^a^	4.20 (510 nm)	82 ^a^	0.82 ^a^
	THF	505	518	497	0.15	5.69	48/166 ^b^	0.31 ^a^
	MeCN	503	512	349	0.01 ^a^	3.62/5.38	18/105 ^b^	0.86 ^a^
**BDP-2**	DCM	552	572	633	0.19	4.06	44/230 ^c^	0.85
	THF	552	568	510	0.30	4.09	49/213 ^b^	0.25
	MeCN	549	571	702	0.01	2.59/5.35	31/138 ^b^	0.81

^a^ Figure taken from work conducted by Wang et al. [39], ^b^ λ_exc_ = 355 nm. ^c^ λ_exc_ = 532 nm.

**Table 2:** ns-TAS lifetimes calculated from kinetic decay curves produced upon excitation of **BDP-1** and **BDP-2**.

	Solvent	abs (nm)	τ_1_ (µs)	τ_2_ (µs)	ɸ_Δ_
**BDP-1**	DCM	507	- ^b^	82 ^a^	0.82 ^a^
	THF	505	48 ^c^	166 ^c^	0.31 ^a^
	MeCN	503	18 ^c^	105 ^c^	0.86 ^a^
**BDP-2**	DCM	552	44 ^d^	230 ^d^	0.85
	THF	552	49 ^c^	213 ^c^	0.25
	MeCN	549	31 ^c^	138 ^c^	0.81

^a^ Figure taken from work conducted by Wang et al. [39], ^b^ Not applicable. ^c^ λ_exc_ = 355 nm. ^d^ λ_exc_ = 532 nm.

**Table 3:** *S. aureus* killing activity of **BDP-1** and **BDP-2** (100 μg·mL^−1^) at varying wavelengths.

Wavelength (nm)	Light Intensity (mW/cm^2^)	Light Dose (mJ/cm^2^)	Log Reduction in CFU·mL^−1 a^	
			BDP-1	BDP-2
370	0.70	2523	5.18	2.68
525	0.05	196	2.11	0.37
550	6.12	22,018	0.61	0.75
570	0.02	64	0.23	0.44

^a^ Log reduction in CFU·mL^−1^ for BDP-treated compared to DMSO controls, where activity in the dark has been subtracted.

## Data Availability

The original contributions presented in this study are included in the article/Supplementary Material. Further inquiries can be directed to the corresponding authors.

## Supplementary Materials

The following supporting information can be downloaded at: https://www.mdpi.com/article/10.3390/molecules30132727/s1, Synthesis of **BDP-1,** Synthesis of **BDP-1a,** Synthesis and Characterization of **BDP-1** and **BDP-1a,** Figure S1. ^1^H NMR for **BDP-1**, CDCl_3_. Figure S2. ^13^C NMR for **BDP-1**, CDCl_3_. Figure S3. ^1^H NMR for **BDP-1a**, CDCl_3_. Figure S5. ^1^H NMR Boc-piperazine propargyl. Figure S6. ^1^H NMR for **BDP-2**, CDCl_3_. Figure S7. ^13^C NMR for **BDP-2**, CDCl_3_. Figure S8. Mass spec. analysis of **BDP-1**. Figure S9. Mass spec. analysis of **BDP-1a**. Figure S10. Mass spec. analysis of **BDP-2**. Figure S11. Emission lifetime for **BDP-1** in MeCN, excited at 510 nm, recorded at 520 nm. Figure S12. Emission lifetime for **BDP-1** in THF, excited at 510 nm, recorded at 520 nm. Figure S14. Emission lifetime for **BDP-2** in MeCN, excited at 510 nm, recorded at 572 nm. Figure S15. Emission lifetime for **BDP-2** in THF, excited at 510 nm, recorded at 572 nm. Figure S16. ns-lifetime decay trace for **BDP-1** in THF (355nm). Figure S17. ns-lifetime decay trace for **BDP-1** in MeCN (355nm). Figure S18. ns-lifetime decay trace of **BDP-2** in THF (355 nm). Figure S19. ns-lifetime decay trace for **BDP-2** in MeCN (355 nm). Figure S20. ns-lifetime decay trace for BDP-2 in DCM (532 nm). Figure S21. Indirect singlet oxygen detection for determination of the singlet oxygen quantum yield of **BDP-2** in MeCN, excited at 528 nm (0 – 180 s). DPBF was used as the singlet oxygen scavenger and the rate of its decrease in absorption at 414 nm monitored to determine the singlet oxygen quantum yield, with **I_2_-BDP** as the standard (ɸ_Δ_ = 0.87). Figure S22. Linear regression analysis of the depletion of absorption features of **BDP-2** and an I_2_-BDP standard, monitored at 414 nm from the indirect singlet oxygen detection using DPBF as a scavenger (12.5 µM) and **BDP-2** as a PS (0.5 µM), with irradiation at 528 nm, in MeCN. Figure S23. Indirect singlet oxygen detection using DPBF as a scavenger (12.5 µM) and **BDP-2** as a PS (0.5 µM), with irradiation at 528 nm, in DCM. Figure S24. Indirect singlet oxygen detection using DPBF as a scavenger (12.5 µM) and **I_2_-BDP** (std) as a PS (0.5 µM), with irradiation at 528 nm, in DCM. Figure S25. Indirect singlet oxygen detection using DPBF as a scavenger (12.5 µM) and **BDP-2** as a PS (0.5 µM), with irradiation at 528 nm, in THF. Figure S26. Indirect singlet oxygen detection using DPBF as a scavenger (12.5 µM) and **I_2_-BDP** (std) as a PS (0.5 µM), with irradiation at 528 nm, in THF. Figure S27. Indirect singlet oxygen detection using DPBF as a scavenger (12.5 µM) and **BDP-2** as a PS (0.5 µM), with irradiation at 528 nm, in MeCN. Figure S28. Indirect singlet oxygen detection using DPBF as a scavenger (12.5 µM) and **I_2_-BDP** (std) as a PS (0.5 µM), with irradiation at 528 nm, in MeCN. Figure S29. Normalized standard curves displaying the decrease in absorption of the BODIPY and DPBF mixtures at 414 nm after irradiation with 528 nm light, in DCM. Figure S30. Normalized standard curves displaying the decrease in absorption of the BODIPY and DPBF mixtures at 414 nm after irradiation with 528 nm light, in THF. Figure S31. Normalized standard curves displaying the decrease in absorption of the BODIPY and DPBF mixtures at 414 nm after irradiation with 528 nm light, in MeCN. Figure S32. A control experiment was conducted by irradiating a solution of DPBF (12.5 µM) in the absence of photosensitiser with irradiation at 528 nm, in MeCN during 180 s. Figure S33. Structure of I2-BDP (std). Figure S33. Scatchard plots of the fluorescence titrations of **BDP-2** (0-40 µM) with BSA (50 µM). Figure S34. Decay trace for the emission signal of BDP-2 at 572 nm, in DCM (following excitation at 375 nm). Fluorescence quantum yield determined using the TCSPC integrating sphere.
