# Supplementary material for: Photophysical Properties and Protein Binding Studies of Piperazine-Substituted Anthracene-BODIPY Dyads for Antimicrobial Photodynamic Therapy
Source: Molecules. 2025 Jun 25;30(13):2727. doi: 10.3390/molecules30132727 (PMC12251241; doi:10.3390/molecules30132727)
Supplement: Supplementary file 1 [file molecules-30-02727-s001.zip › molecules-3684698-supplementary.pdf]

Supporting Information

Photophysical Properties and Protein Binding Studies of Piperazine-Substituted Anthracene-BODIPY Dyads for Antimicrobial Photodynamic Therapy

Stephen O'Sullivan [1], Leila Tabrizi [1], Kaja Turzańska [2], Ian P. Clark [3], Deirdre Fitzgerald-Hughes [2,*] and Mary T. Pryce [1,*]

[1] School of Chemical Sciences, Dublin City University, D09W6Y4 Dublin, Ireland

[2] Department of Clinical Microbiology, Royal College of Surgeons in Ireland, RCSI Education and Research, Beaumont Hospital, Beaumont, D09YD60 Dublin, Ireland

[3]Central Laser Facility, Research Complex at Harwell, STFC Rutherford Appleton Laboratory, Harwell Campus, Didcot OX11 0QX, Oxfordshire, UK

[*]Email: mary.pryce@dcu.ie, dfitzgeraldhughes@rcsi.ie

**Contents**	Pages
Synthesis of **BDP-1**	3
Synthesis of **BDP-1a**	3
Synthesis and Characterization of **BDP-1** and **BDP-1a**	4
Figure S1. ^{1}H NMR for **BDP-1**, $CDCl_3$.	5
Figure S2. ^{13}C NMR for **BDP-1**, $CDCl_3$.	5
Figure S3. ^{1}H NMR for **BDP-1a**, $CDCl_3$.	6
Figure S4. ^{13}C NMR for **BDP-1a**, $CDCl_3$.	6
Figure S5. ^{1}H NMR Boc-piperazine propargyl.	7
Figure S6. ^{1}H NMR for **BDP-2**, $CDCl_3$.	7
Figure S7. ^{13}C NMR for **BDP-2**, $CDCl_3$.	8
Figure S8. Mass spec. analysis of **BDP-1**.	8
Figure S9. Mass spec. analysis of **BDP-1a**.	9
Figure S10. Mass spec. analysis of **BDP-2**.	9
Figure S11. Emission lifetime for **BDP-1** in MeCN, excited at 510 nm, recorded at 520 nm.	10
Figure S12. Emission lifetime for **BDP-1** in THF, excited at 510 nm, recorded at 520 nm.	10
Figure S13. Emission lifetime for **BDP-2** in DCM, excited at 510 nm, recorded at 572 nm.	11
Figure S14. Emission lifetime for **BDP-2** in MeCN, excited at 510 nm, recorded at 572 nm.	12
Figure S15. Emission lifetime for **BDP-2** in THF, excited at 510 nm, recorded at 572 nm.	12

Figure S16. ns-lifetime decay trace for **BDP-1** in THF (355nm).	13
Figure S17. ns-lifetime decay trace for **BDP-1** in MeCN (355nm).	13
Figure S18. ns-lifetime decay trace of **BDP-2** in THF (355 nm).	13
Figure S19. ns-lifetime decay trace for **BDP-2** in MeCN (355 nm).	14
Figure S20. ns-lifetime decay trace for BDP-2 in DCM (532 nm).	14
Figure S21. Indirect singlet oxygen detection for determination of the singlet oxygen quantum yield of **BDP-2** in MeCN, excited at 528 nm (0 – 180 s). DPBF was used as the singlet oxygen scavenger and the rate of its decrease in absorption at 414 nm monitored to determine the singlet oxygen quantum yield, with **I_2-BDP** as the standard (ϕ_Δ = 0.87).	15
Figure S22. Linear regression analysis of the depletion of absorption features of **BDP-2** and an I_2-BDP standard, monitored at 414 nm from the indirect singlet oxygen detection using DPBF as a scavenger (12.5 μM) and **BDP-2** as a PS (0.5 μM), with irradiation at 528 nm, in MeCN.	16
Figure S23. Indirect singlet oxygen detection using DPBF as a scavenger (12.5 μM) and **BDP-2** as a PS (0.5 μM), with irradiation at 528 nm, in DCM.	16
Figure S24. Indirect singlet oxygen detection using DPBF as a scavenger (12.5 μM) and **I_2-BDP** (std) as a PS (0.5 μM), with irradiation at 528 nm, in DCM.	16
Figure S25. Indirect singlet oxygen detection using DPBF as a scavenger (12.5 μM) and **BDP-2** as a PS (0.5 μM), with irradiation at 528 nm, in THF.	17
Figure S26. Indirect singlet oxygen detection using DPBF as a scavenger (12.5 μM) and **I_2-BDP** (std) as a PS (0.5 μM), with irradiation at 528 nm, in THF.	17
Figure S27. Indirect singlet oxygen detection using DPBF as a scavenger (12.5 μM) and **BDP-2** as a PS (0.5 μM), with irradiation at 528 nm, in MeCN.	17
Figure S28. Indirect singlet oxygen detection using DPBF as a scavenger (12.5 μM) and **I_2-BDP** (std) as a PS (0.5 μM), with irradiation at 528 nm, in MeCN.	18
Figure S29. Normalised standard curves displaying the decrease in absorption of the BODIPY and DPBF mixtures at 414 nm after irradiation with 528 nm light, in DCM.	18
Figure S30. Normalised standard curves displaying the decrease in absorption of the BODIPY and DPBF mixtures at 414 nm after irradiation with 528 nm light, in THF.	19
Figure S31. Normalised standard curves displaying the decrease in absorption of the BODIPY and DPBF mixtures at 414 nm after irradiation with 528 nm light, in MeCN.	19
Figure S32. A control experiment was conducted by irradiating a solution of DPBF (12.5 μM) in the absence of photosensitiser with irradiation at 528 nm, in MeCN during 180 s.	20
Figure S33. Structure of I2-BDP (std)	20
Figure S33. Scatchard plots of the fluorescence titrations of **BDP-2** (0-40 μM) with BSA (50 μM).	21
Figure S34. Decay trace for the emission signal of BDP-2 at 572 nm, in DCM (following excitation at 375 nm). Fluorescence quantum yield determined using the TCSPC integrating sphere.	21

Synthesis of BDP-1

9-anthracenecarboxaldehyde (516 mg, 2.5 mmol) and 2,4-dimethyl-1,H-pyrrole (0.51 mL, 5.0 mmol) were added to anhydrous dichloromethane (DCM, 30 mL). Magnetic stirring was commenced and 1 drop of trifluoroacetic acid (TFA) was added to the reaction mixture as a catalyst and allowed to stir overnight at room temperature. 2,3-Dichloro-5,6-dicyano-1,4-benzoquinone (DDQ) (568 mg, 2.5 mmol) was then added and allowed to stir for two hours. After this triethylamine (TEA) (6 mL, 43.0 mmol) was added, followed shortly by dropwise addition of borontrifluoride diethyletherate ($BF_3{\cdot}Et_2O$) (3 mL, 24.3 mmol). The reaction was the left stirring overnight at room temperature. The resulting crude mixture was then washed with saturated sodium carbonate (Na_2CO_3, 30 mL (×3)) solution and dried over magnesium sulphate ($MgSO_4$). The product was then purified by silica column chromatography, with a mobile phase of DCM and hexane (1:1), to afford an orange solid (37%). %). ^{1}H NMR (600 MHz, $CDCl_3$): δ = 8.56 (s, 1H), 8.02 (d, 2H), 7.89 (d, 2H), 7.47 (t, 2H), 7.41 (t, 2H), 5.88 (s, 2H), 2.60 (s, 6H), 0.62 (s, 6H). ^{13}C NMR ($CDCl_3$): δ = 155.95, 143.08, 139.14, 131.48, 129.86, 128.49, 128.41, 127.11, 125.89, 125.29, 121.32, 14.86, 13.48. MS: m/z: 447.1828 $[M + Na]^+$.

Synthesis of BDP-1a

BDP-1 (200 mg, 0.47 mmol) and iodine (I_2) (297 mg, 1.17 mmol) were dissolved in 30 mL of ethanol (EtOH) and anhydrous dichloromethane (DCM, 3 mL) in a round-bottomed flask. Iodic acid (HIO_3) (205 mg, 1.17 mmol) was dissolved in deionised water (2.5 mL) and added to the mixture with magnetic stirring. The mixture was stirred at room temperature overnight at room temperature. The crude mixture was then dried under reduced pressure and redissolved in DCM (20 mL). The crude mixture was then washed with saturated aqueous sodium thiosulfate ($Na_2S_2O_3$, 30 mL (×3)) solution to convert the organic-soluble I_2 to water soluble iodide (I^-). The organic layer was then dried over $MgSO_4$ and gravity filtered. The filtrate was then dried under reduced pressure to afford a red solid (91%). ^{1}H NMR (600 MHz, $CDCl_3$): δ = 8.62 (s,

1H), 8.05 (d, 2H, ^3J = 8.5 Hz), 7.81 (d, 2H, ^3J = 8.6 Hz), 7.50 (t, 2H, ^3J = 7.5 Hz), 7.43 (t, 2H, ^3J = 7.6 Hz), 2.71 (s, 6H), 0.66 (s, 6H). ^{13}C NMR ($CDCl_3$): δ = 157.21, 145.26, 132.30, 131.45, 129.66, 129.04, 128.68, 127.80, 127.56, 126.10, 124.86, 16.32, 15.94. MS: m/z: 677.0138 [M + H]$^+$.

Synthesis and Characterization of **BDP-1** and **BDP-1a**

The synthesis of **BDP-1** followed a previously reported methods with slight modifications, resulting in an orange solid and a yield of 37%. The ^{1}H NMR spectrum of **BDP-1** confirmed its successful synthesis (Figure S1). The proton signal observed at 8.56 ppm (singlet, 1H) in the ^{1}H NMR spectrum corresponds to one of the aromatic protons of the anthracene moiety that is conjugated to the BODIPY core. This chemical shift is consistent with the deshielded environment expected for an inner proton on the central ring of anthracene, especially in the context of extended conjugation with the electron-withdrawing BODIPY unit. The two methyl groups on the pyrrole ring are observed as singlets at δ 2.60 ppm, and the meso-position methyl groups appear at δ 0.62 ppm.

BDP-1a was synthesised via iodination of **BDP-1** using iodine and iodic acid, yielding a red solid in a high yield of 91%. The ^{1}H NMR (Figure S3) confirmed the substitution reaction had occurred as expected. Moreover, the formation of **BDP-1** and **BDP-1a** is confirmed by the parent peaks observed in the mass spectra at m/z = 447.1828 ([M + Na]$^+$) and m/z = 677.0138 ([M + H]$^+$), respectively (Figures S8 and S9, Supporting Information).

NMRs

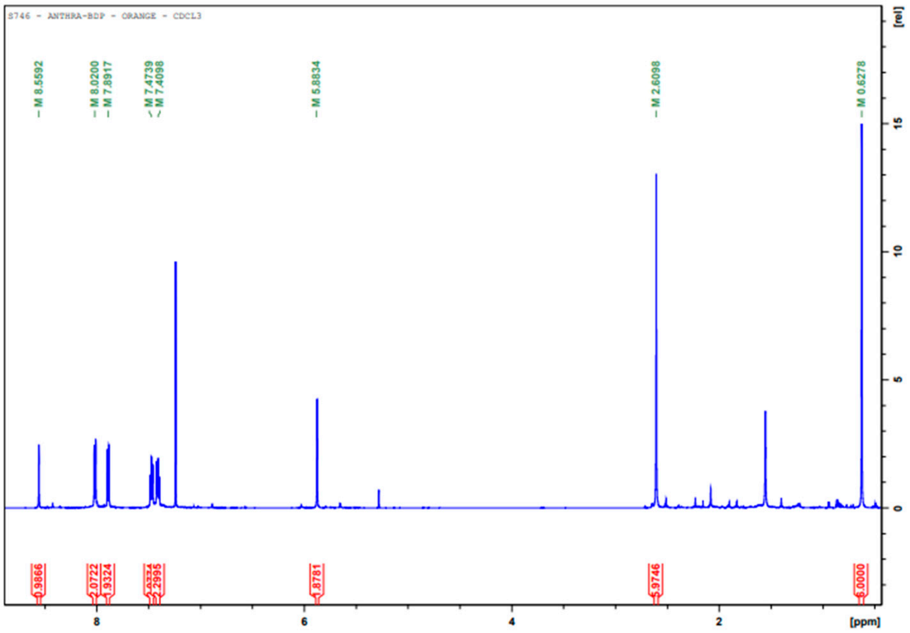

Figure S1. ^{1}H NMR for **BDP-1**, $CDCl_3$.

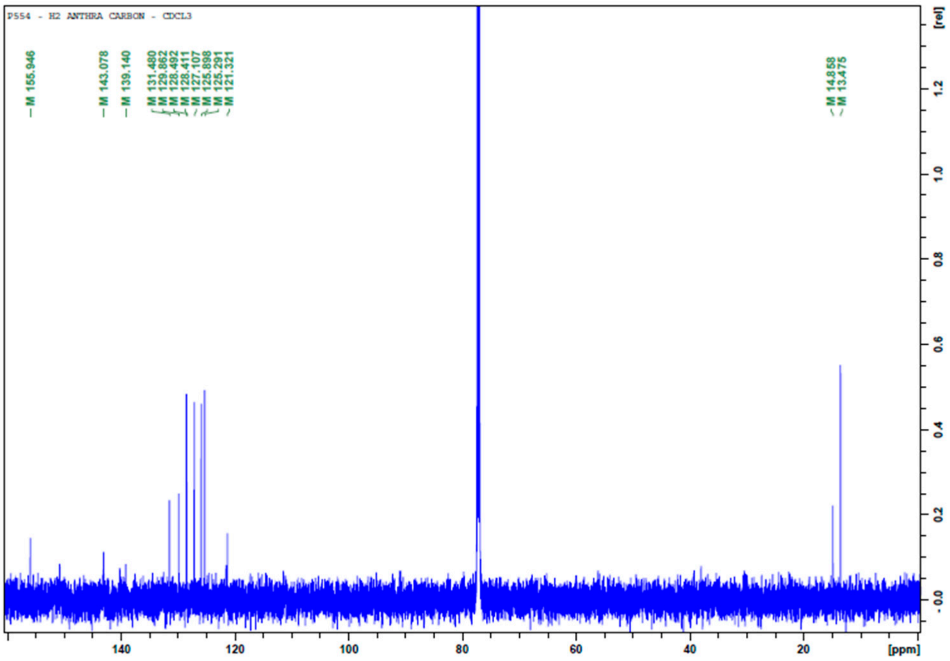

Figure S2. ^{13}C NMR for **BDP-1**, $CDCl_3$.

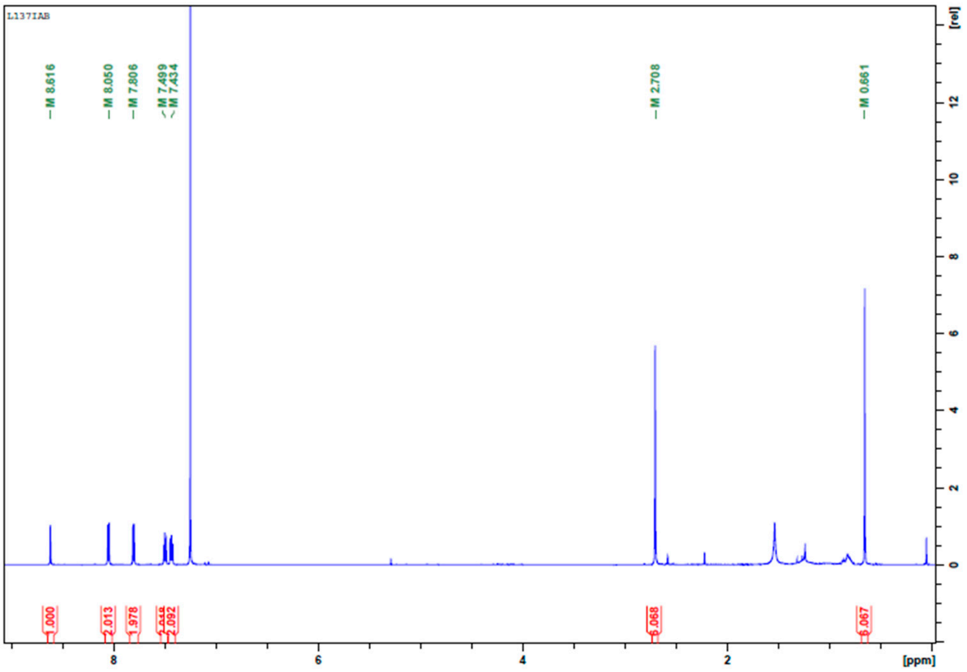

Figure S3. ^{1}H NMR for **BDP-1a**, $CDCl_3$.

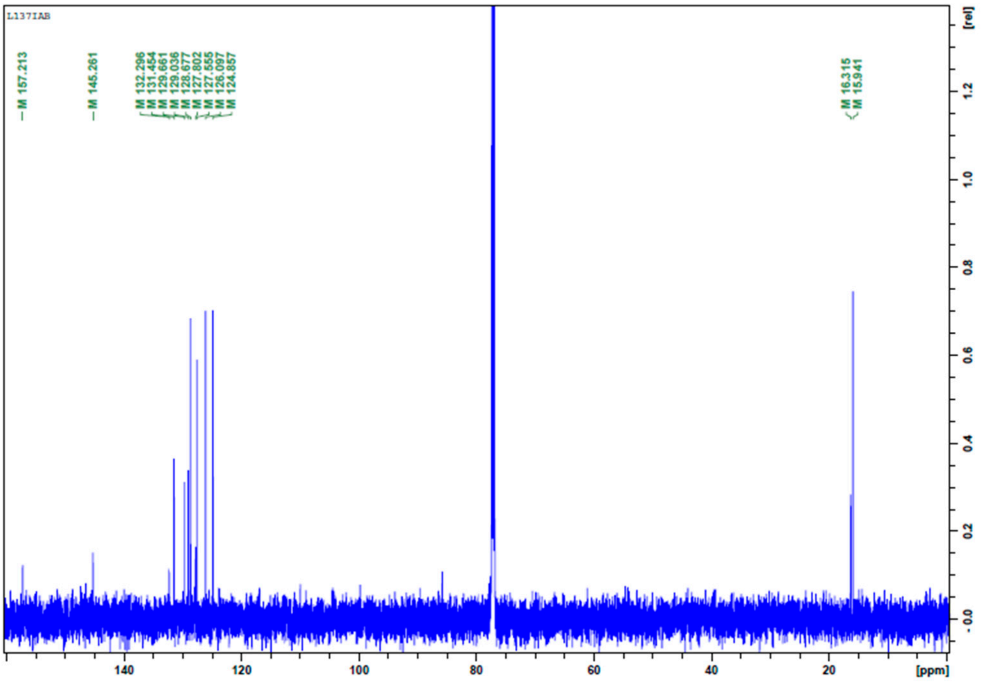

Figure S4. ^{13}C NMR for **BDP-1a**, $CDCl_3$.

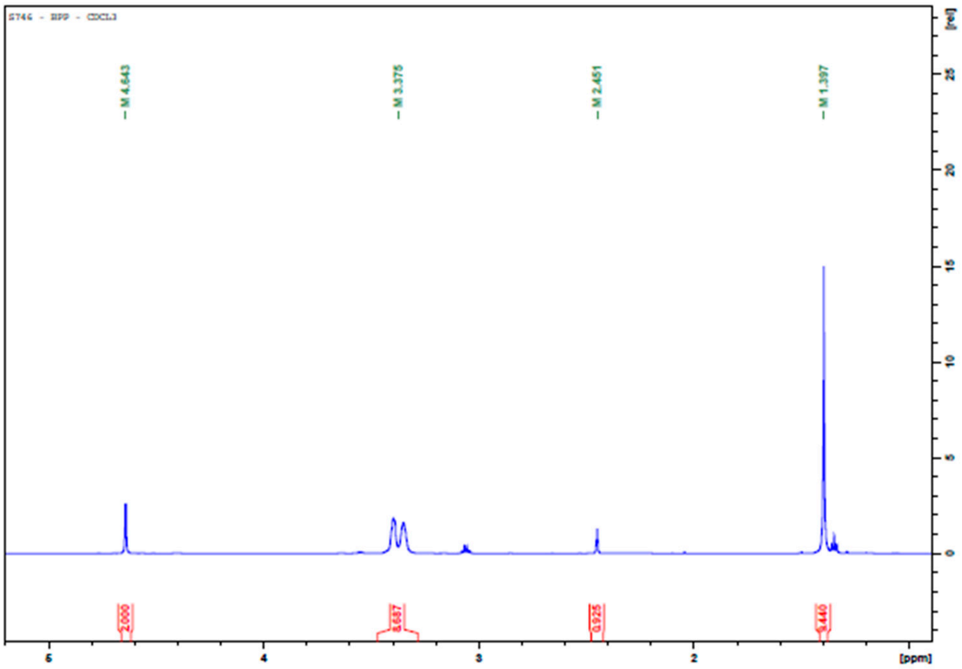

Figure S5. ^{1}H NMR Boc-piperazine propargyl.

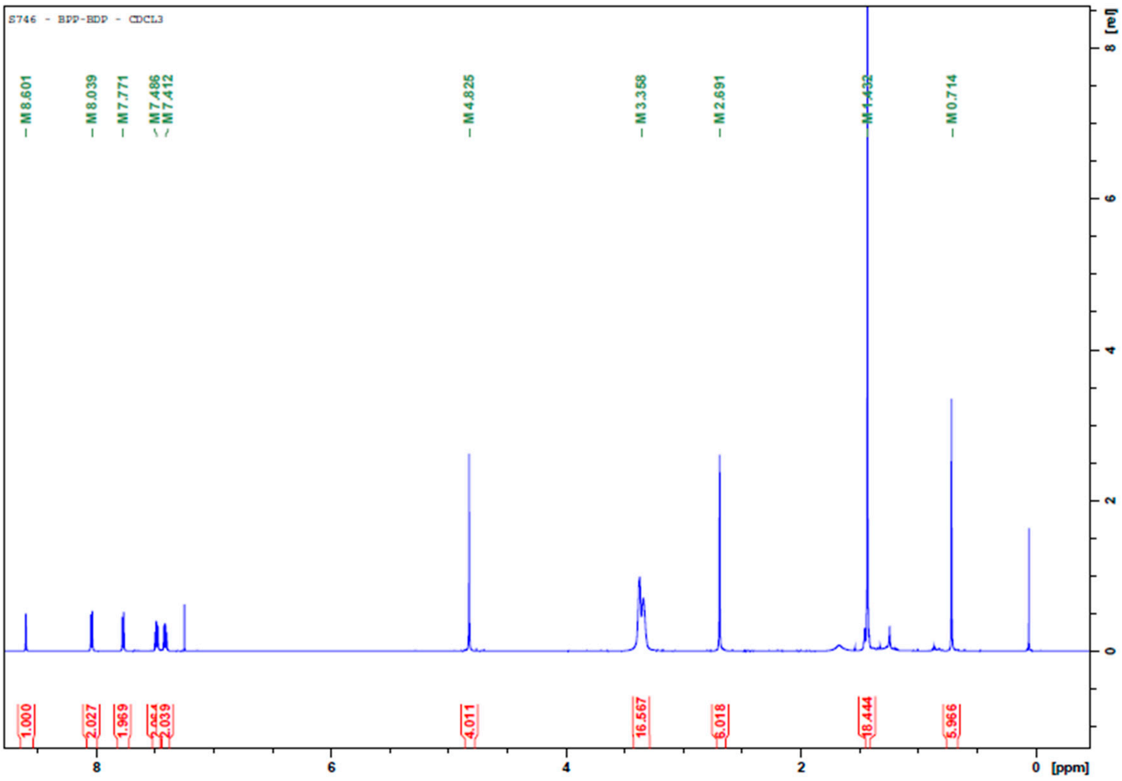

Figure S6. ^{1}H NMR for **BDP-2**, $CDCl_3$.

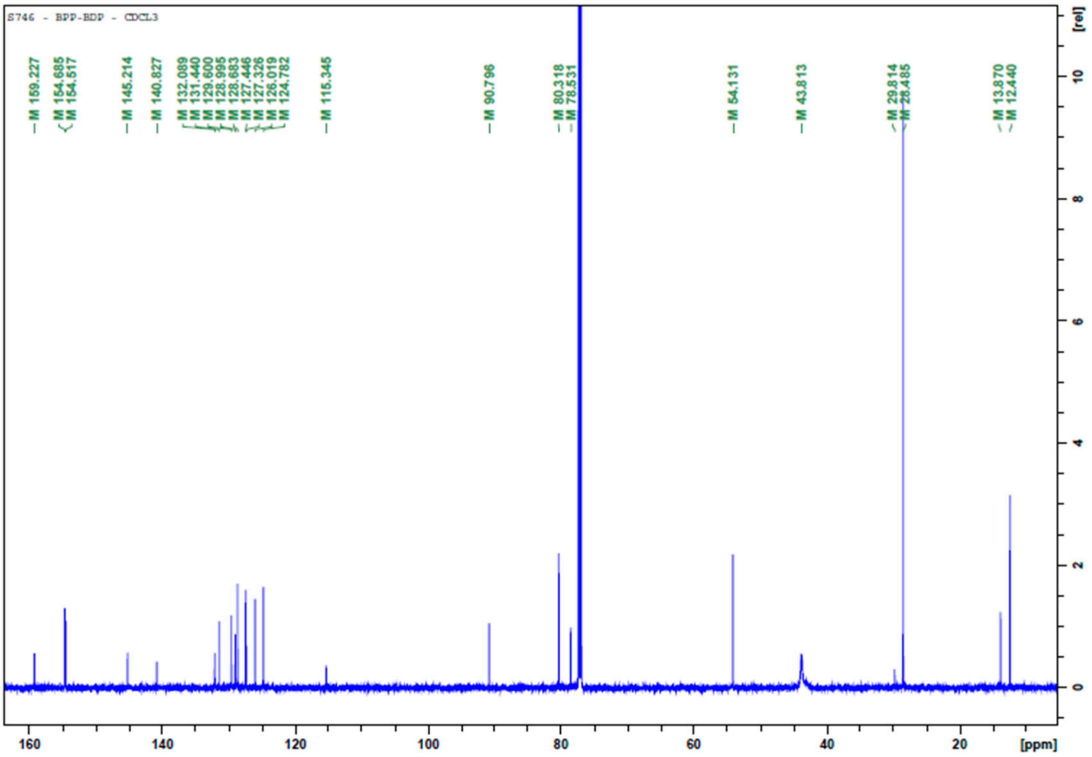

Figure S7. ^{13}C NMR for **BDP-2**, $CDCl_3$.

Mass Spec

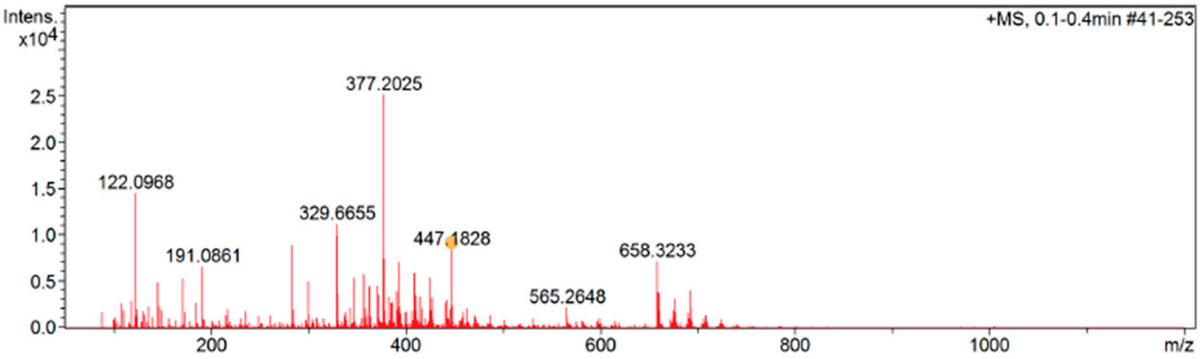

Meas. m/z	#	Ion Formula	m/z	err [ppm]	mSigma	# mSigma	Score	rdb	e⁻ Conf	N-Rule
447.1828	1	C27H23BF2N2Na	447.1815	-1.9	20.5	1	100.00	16.5	even	ok

Figure S8. Mass spec. analysis of **BDP-1**.

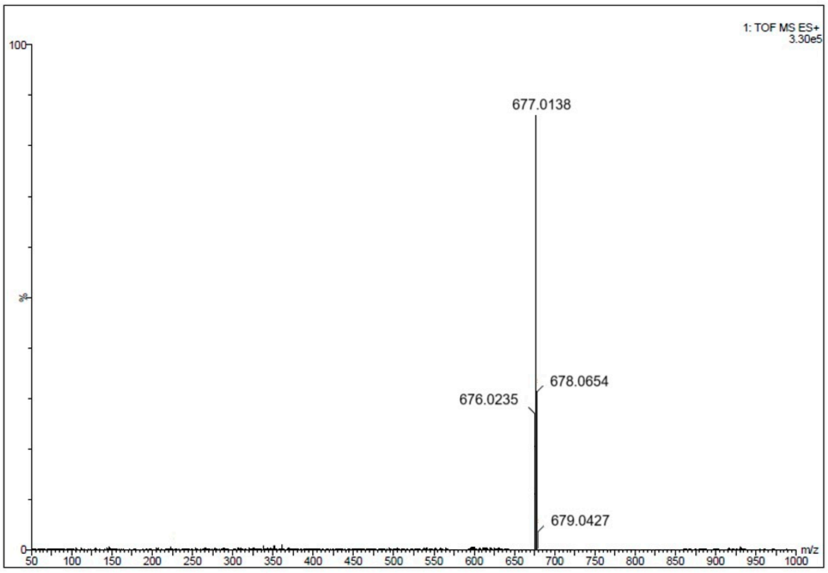

Figure S9. Mass spec. analysis of **BDP-1a.**

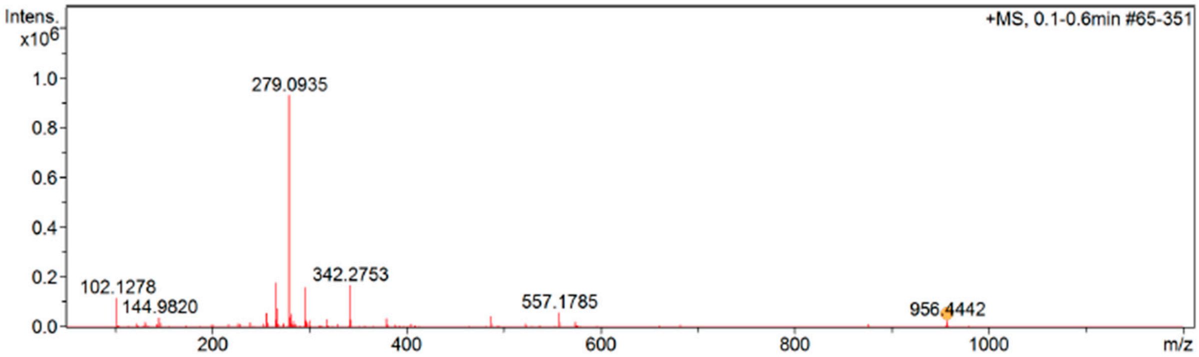

Meas. m/z	#	Ion Formula	m/z	err [ppm]	mSigma	# mSigma	Score	rdb	e⁻ Conf	N-Rule
956.4442	1	C53H59BF2N6O8	956.4450	1.7	22.5	1	100.00	27.0	odd	ok

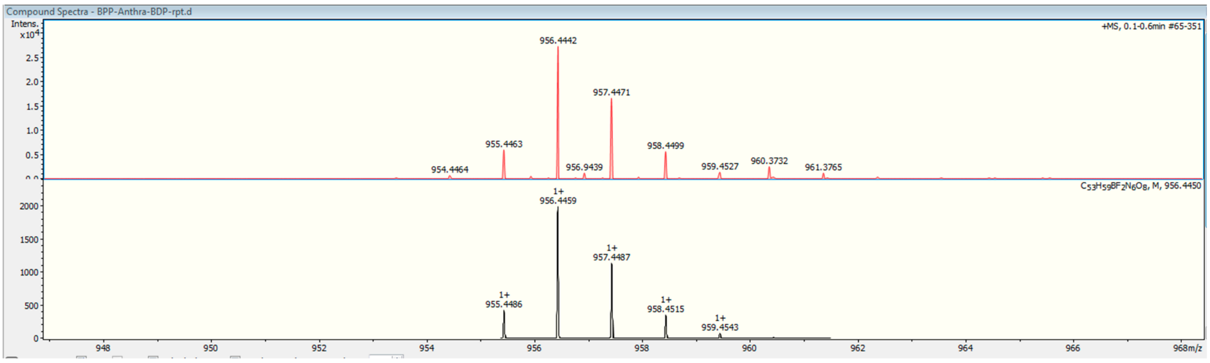

Figure S10. Mass spec. analysis of **BDP-2**.

Photophysical study

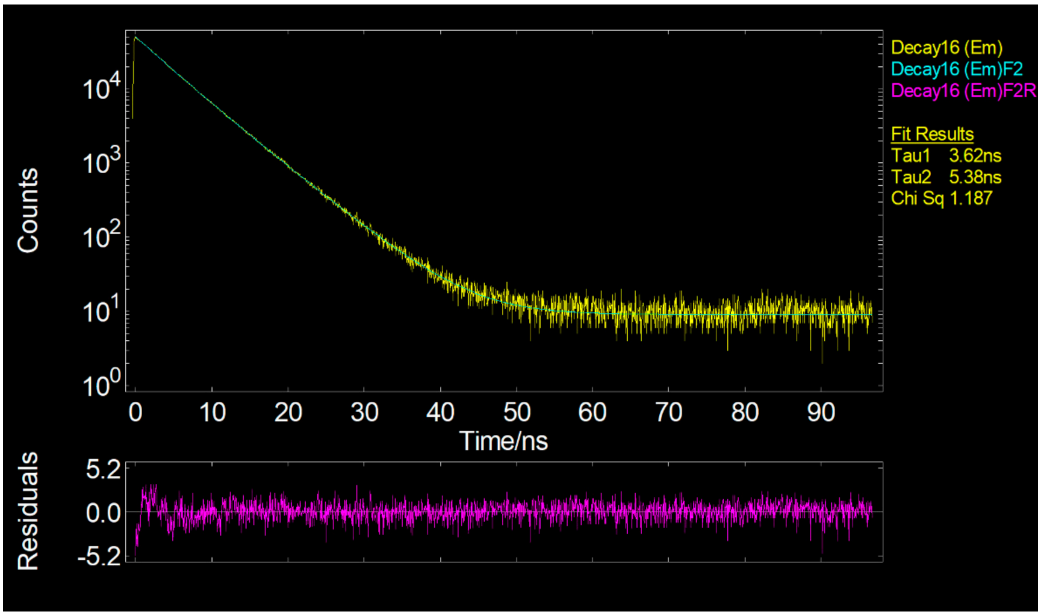

Figure S11. Emission lifetime for **BDP-1** in MeCN, excited at 510 nm, recorded at 520 nm.

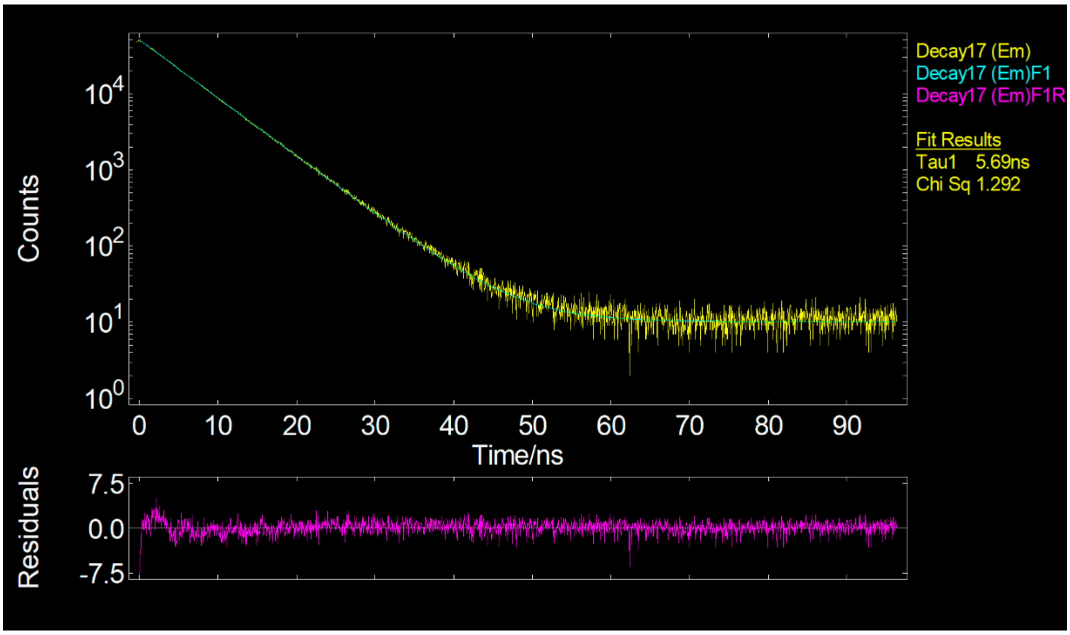

Figure S12. Emission lifetime for **BDP-1** in THF, excited at 510 nm, recorded at 520 nm.

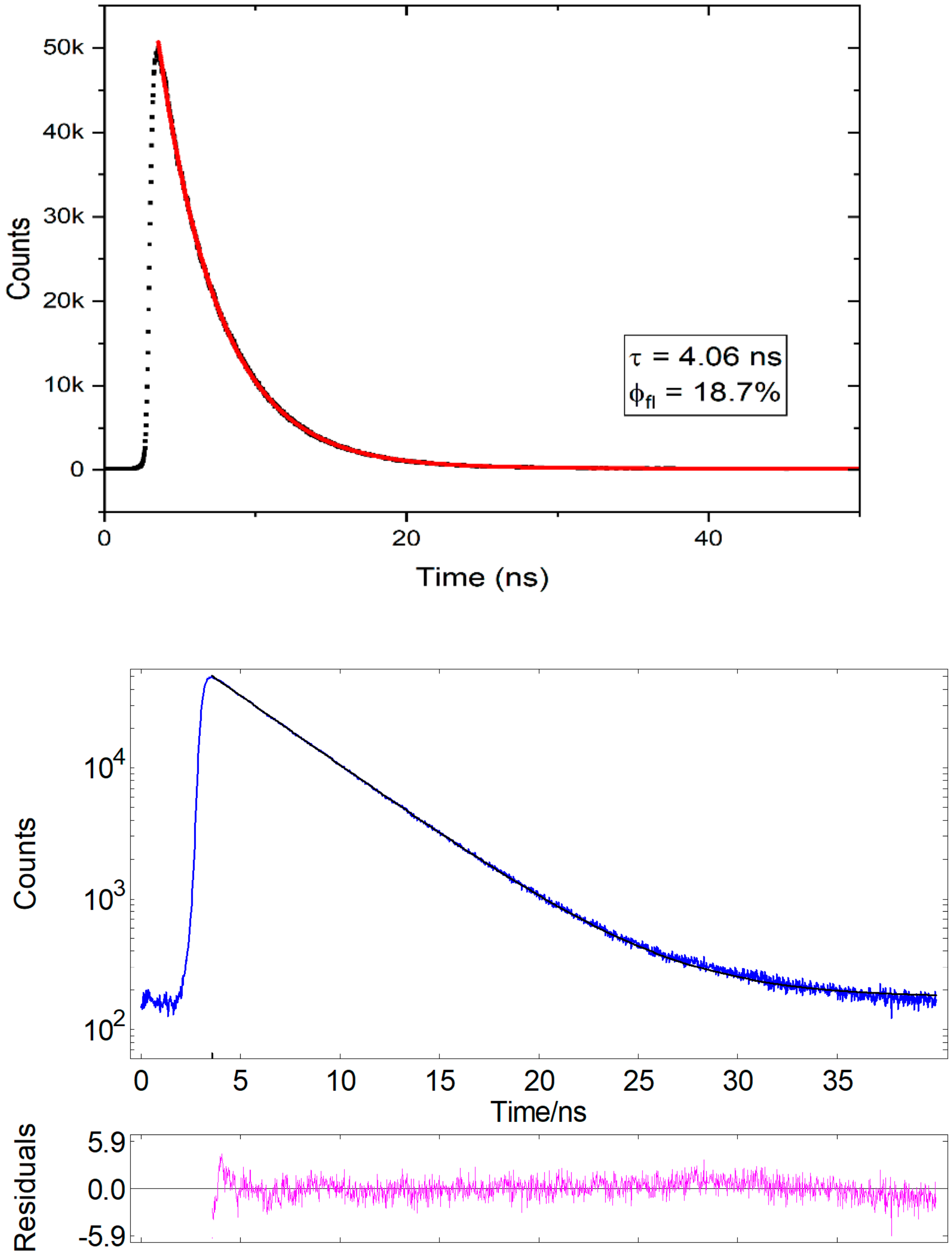

Figure S13. Emission lifetime for **BDP-2** in DCM, excited at 510 nm, recorded at 572 nm.

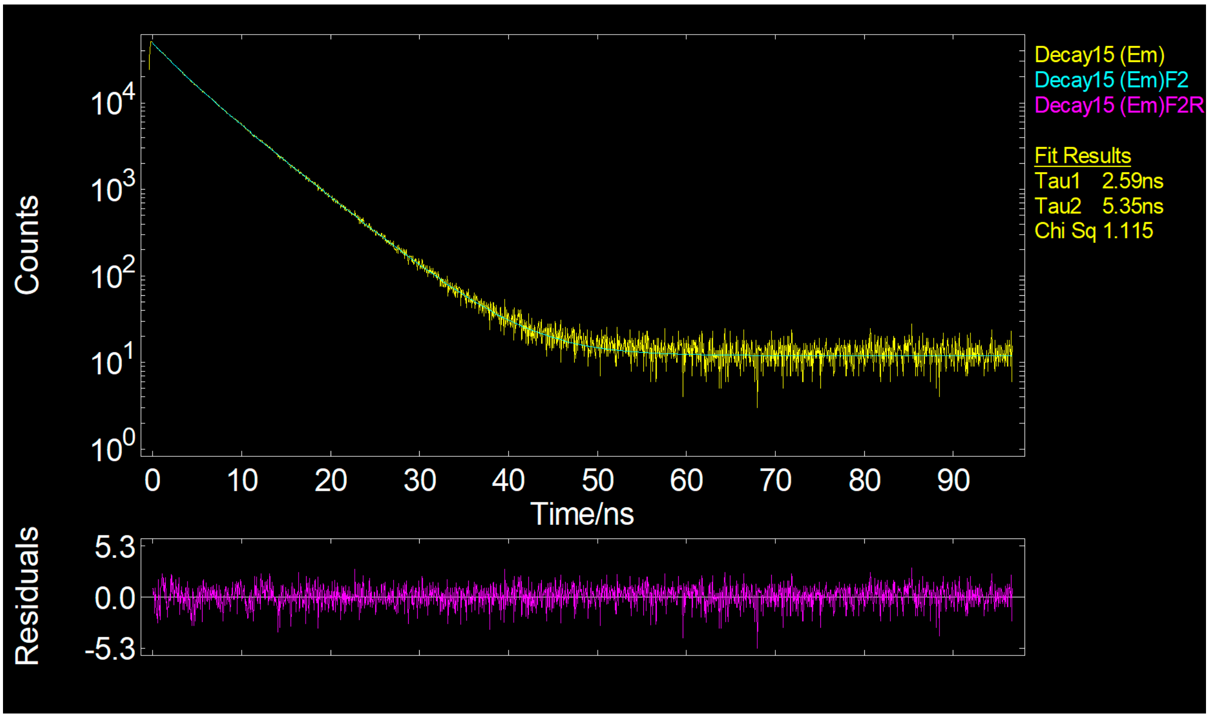

Figure S14. Emission lifetime for **BDP-2** in MeCN, excited at 510 nm, recorded at 572 nm.

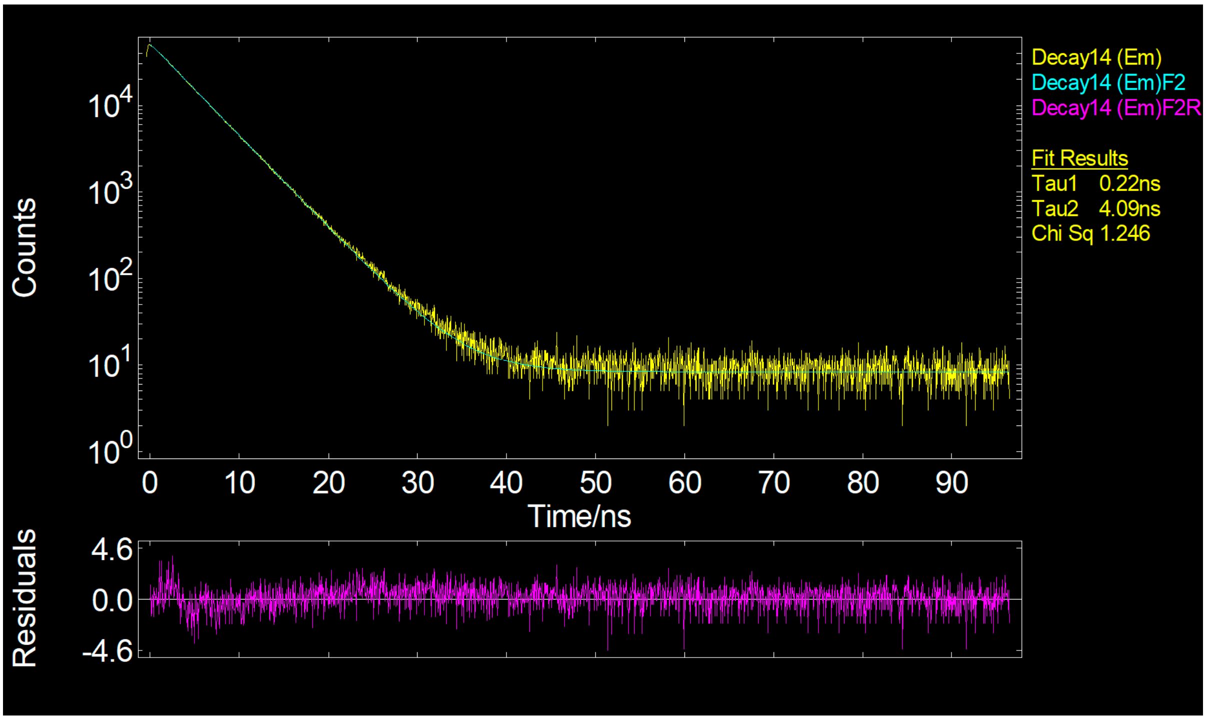

Figure S15. Emission lifetime for **BDP-2** in THF, excited at 510 nm, recorded at 572 nm.

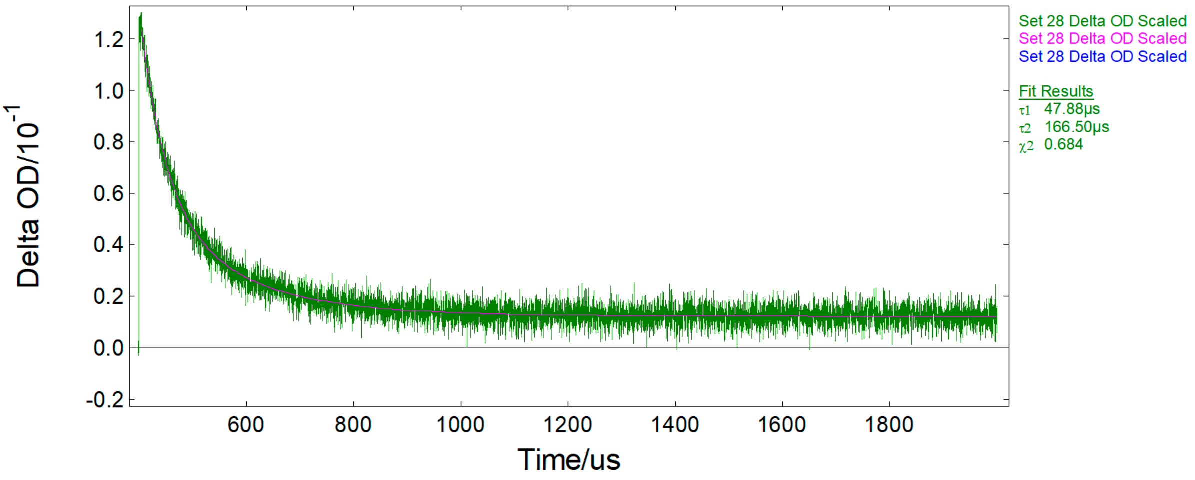

Figure S16. ns-lifetime decay trace for **BDP-1** in THF (355nm).

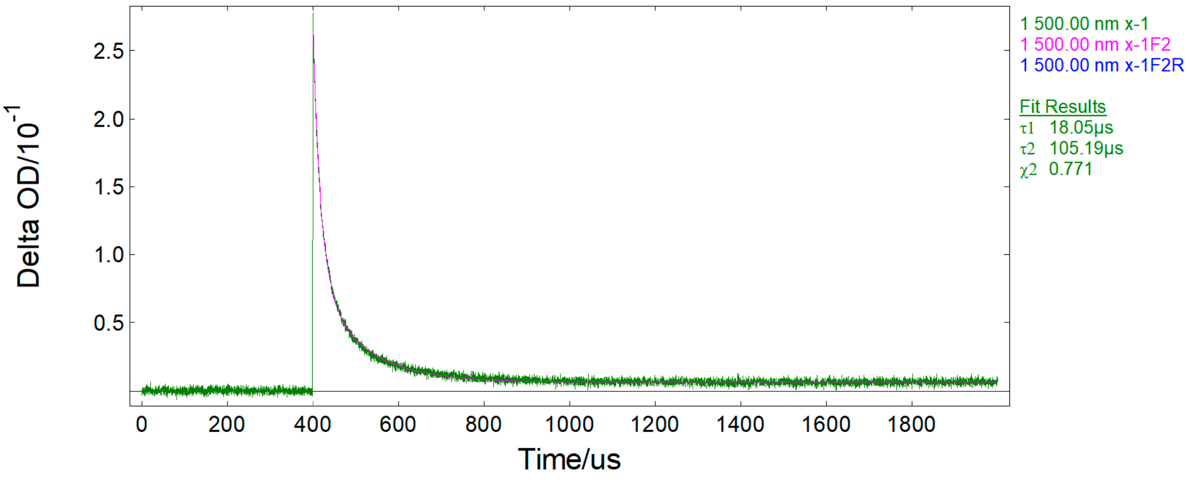

Figure S17. ns-lifetime decay trace for **BDP-1** in MeCN (355nm).

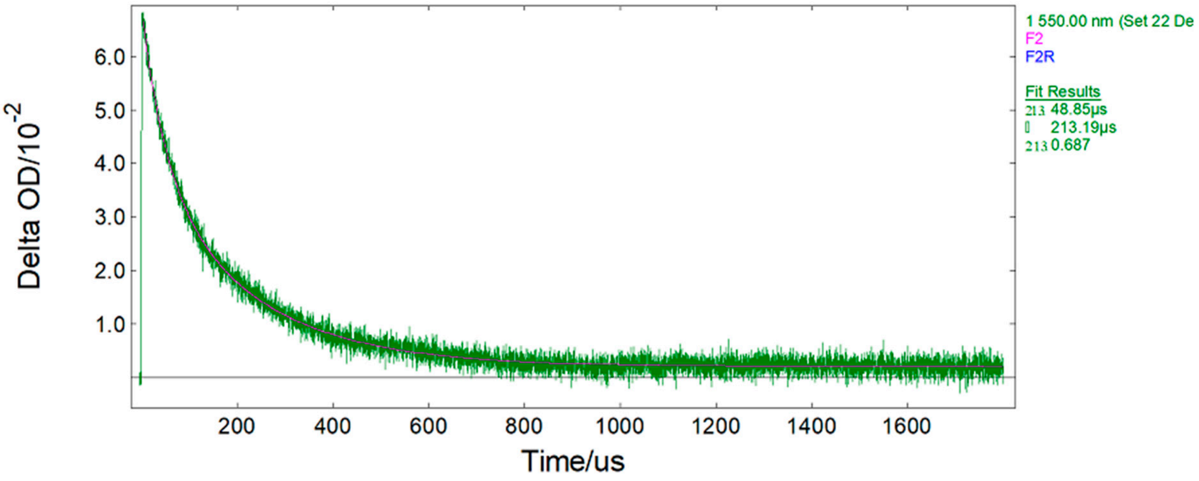

Figure S18. ns-lifetime decay trace of **BDP-2** in THF (355 nm).

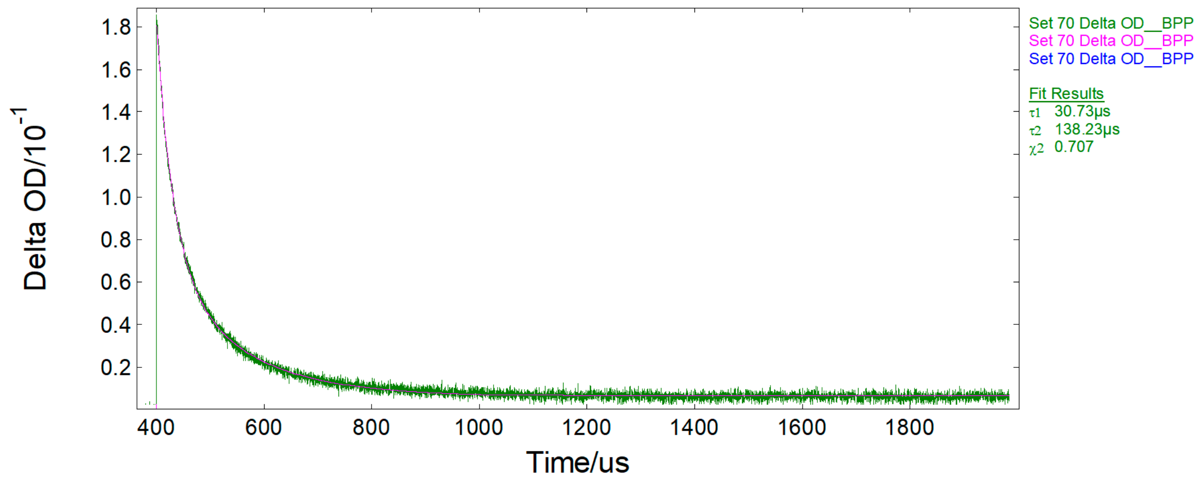

Figure S19. ns-lifetime decay trace for **BDP-2** in MeCN (355 nm).

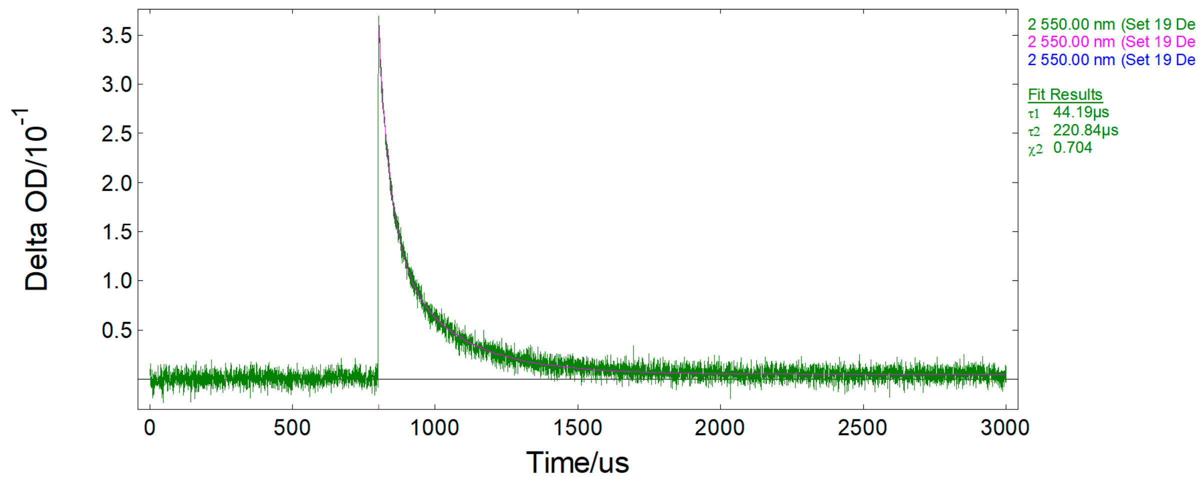

Figure S20. ns-lifetime decay trace for **BDP-2** in DCM (532 nm).

Singlet oxygen detection

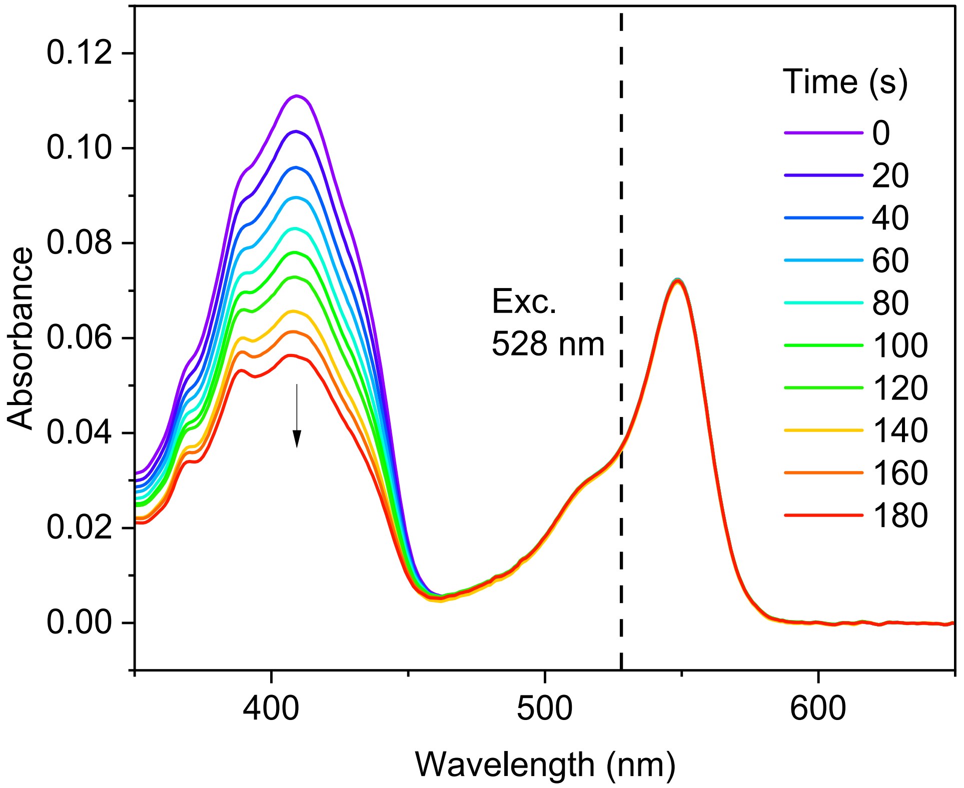

Figure S21. Indirect singlet oxygen detection for determination of the singlet oxygen quantum yield of **BDP-2** in MeCN, excited at 528 nm (0 – 180 s). DPBF was used as the singlet oxygen scavenger and the rate of its decrease in absorption at 414 nm monitored to determine the singlet oxygen quantum yield, with **I$_2$-BDP** as the standard (ϕ_Δ = 0.87).

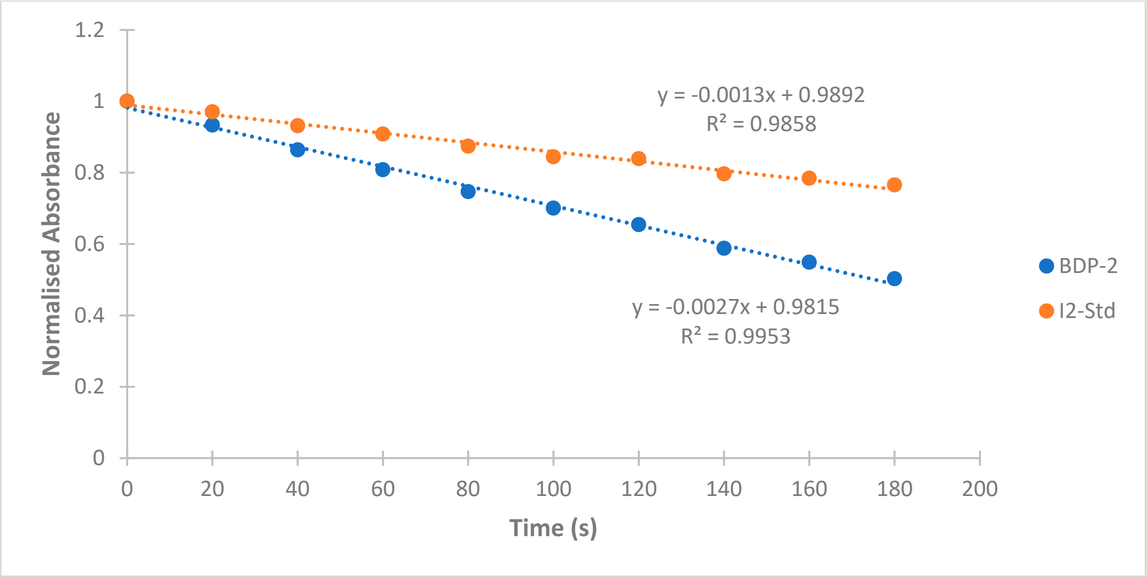

Figure S22. Linear regression analysis of the depletion of absorption features of **BDP-2** and an I_2-BDP standard, monitored at 414 nm from the indirect singlet oxygen detection using DPBF as a scavenger (12.5 µM) and **BDP-2** as a PS (0.5 µM), with irradiation at 528 nm, in MeCN.

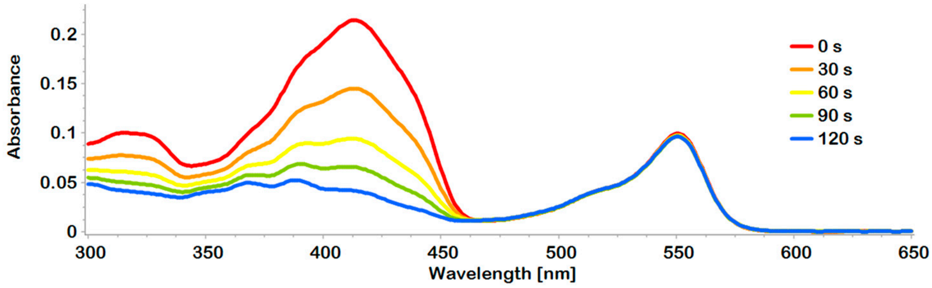

Figure S23. Indirect singlet oxygen detection using DPBF as a scavenger (12.5 µM) and **BDP-2** as a PS (0.5 µM), with irradiation at 528 nm, in DCM.

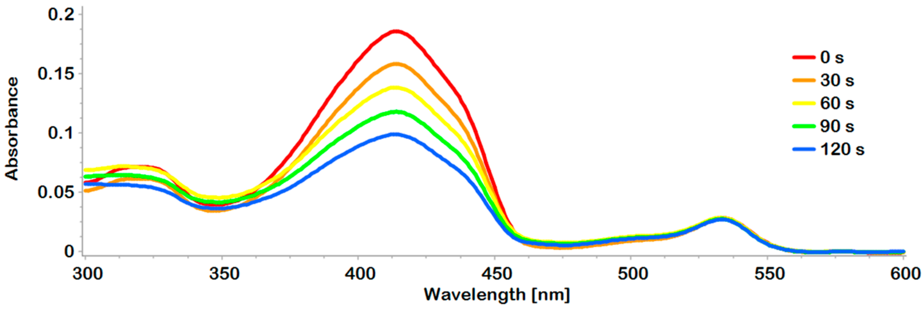

Figure S24. Indirect singlet oxygen detection using DPBF as a scavenger (12.5 µM) and **I_2-BDP** (std) as a PS (0.5 µM), with irradiation at 528 nm, in DCM.

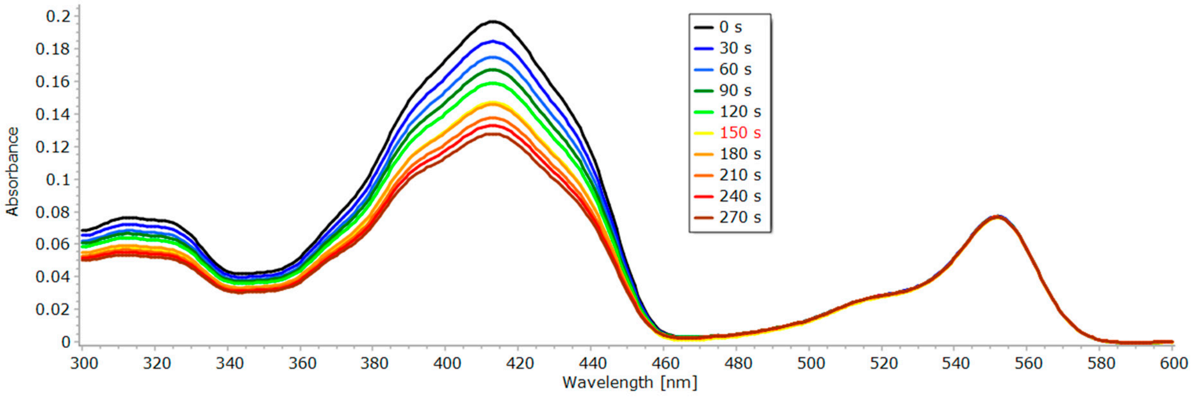

Figure S25. Indirect singlet oxygen detection using DPBF as a scavenger (12.5 µM) and **BDP-2** as a PS (0.5 µM), with irradiation at 528 nm, in THF.

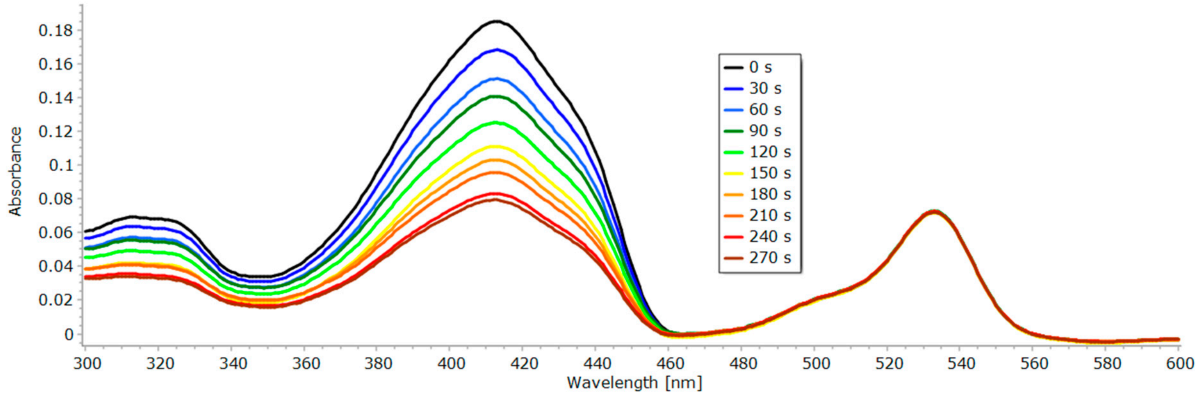

Figure S26. Indirect singlet oxygen detection using DPBF as a scavenger (12.5 µM) and **I_2-BDP** (std) as a PS (0.5 µM), with irradiation at 528 nm, in THF.

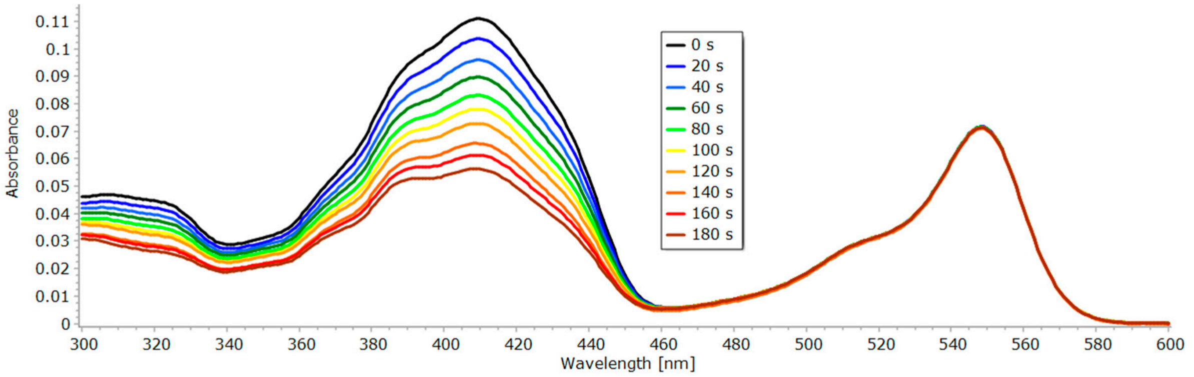

Figure S27. Indirect singlet oxygen detection using DPBF as a scavenger (12.5 µM) and **BDP-2** as a PS (0.5 µM), with irradiation at 528 nm, in MeCN.

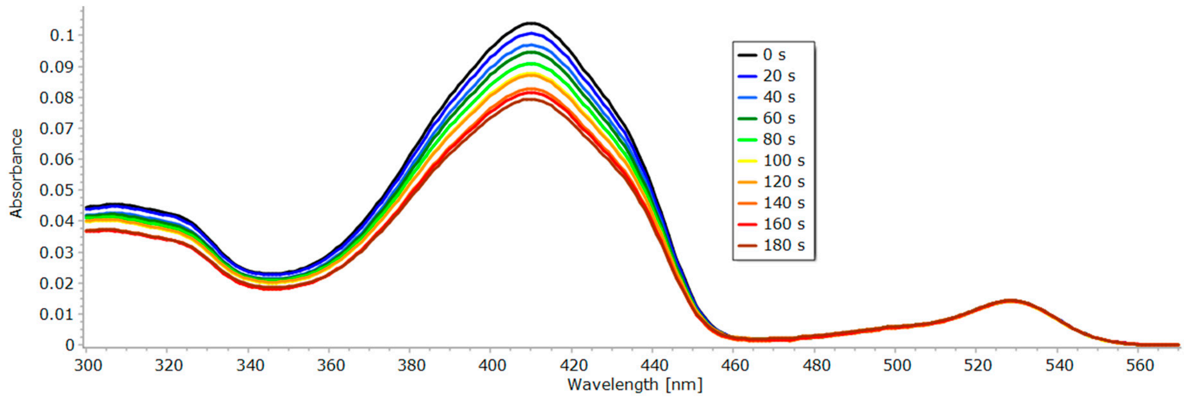

Figure S28. Indirect singlet oxygen detection using DPBF as a scavenger (12.5 μM) and **I_2-BDP** (std) as a PS (0.5 μM), with irradiation at 528 nm, in MeCN.

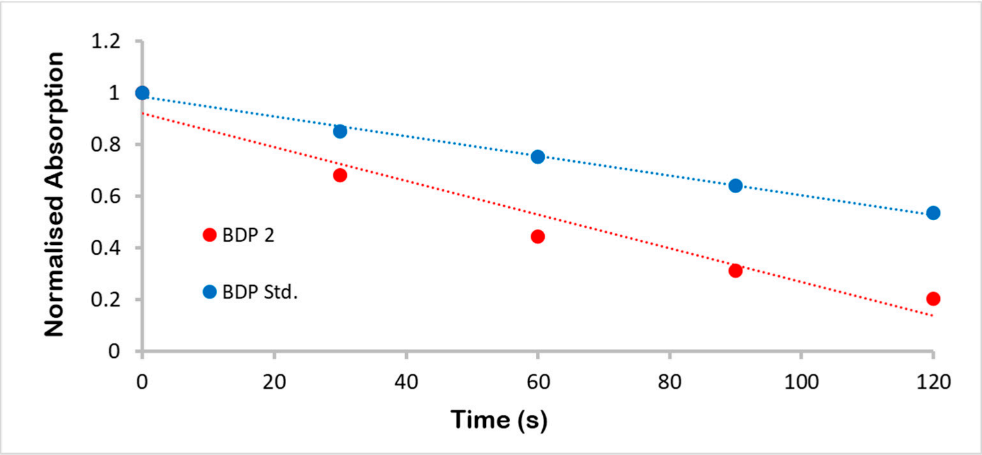

Figure S29. Normalised standard curves displaying the decrease in absorption of the BODIPY and DPBF mixtures at 414 nm after irradiation with 528 nm light, in DCM.

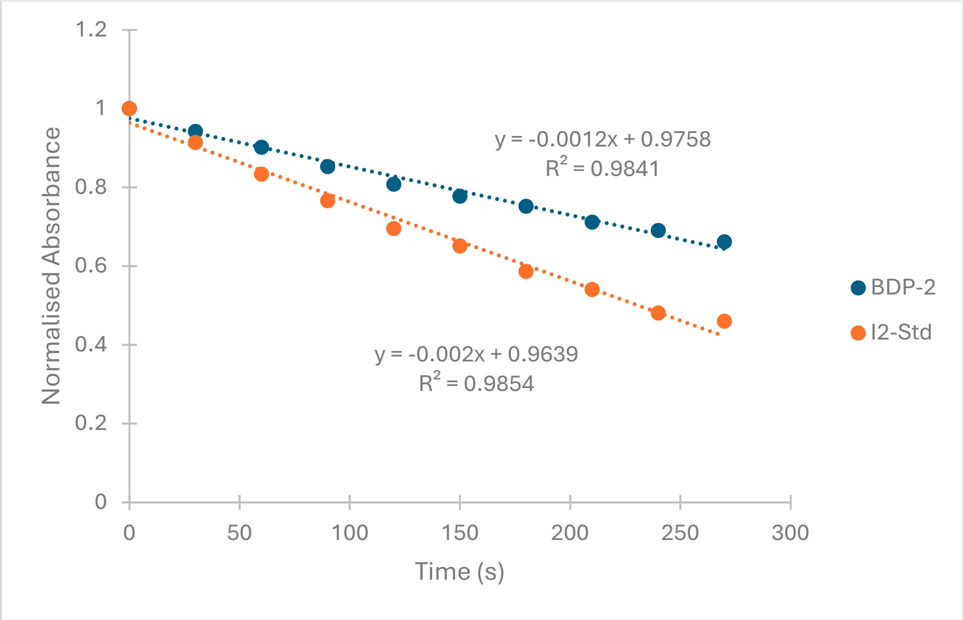

Figure S30. Normalised standard curves displaying the decrease in absorption of the BODIPY and DPBF mixtures at 414 nm after irradiation with 528 nm light, in THF.

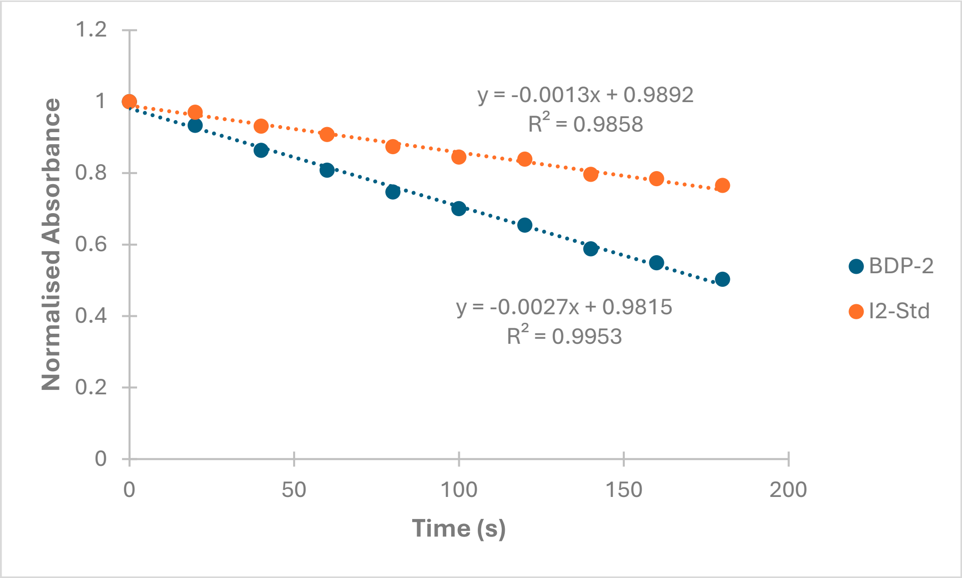

Figure S31. Normalised standard curves displaying the decrease in absorption of the BODIPY and DPBF mixtures at 414 nm after irradiation with 528 nm light, in MeCN.

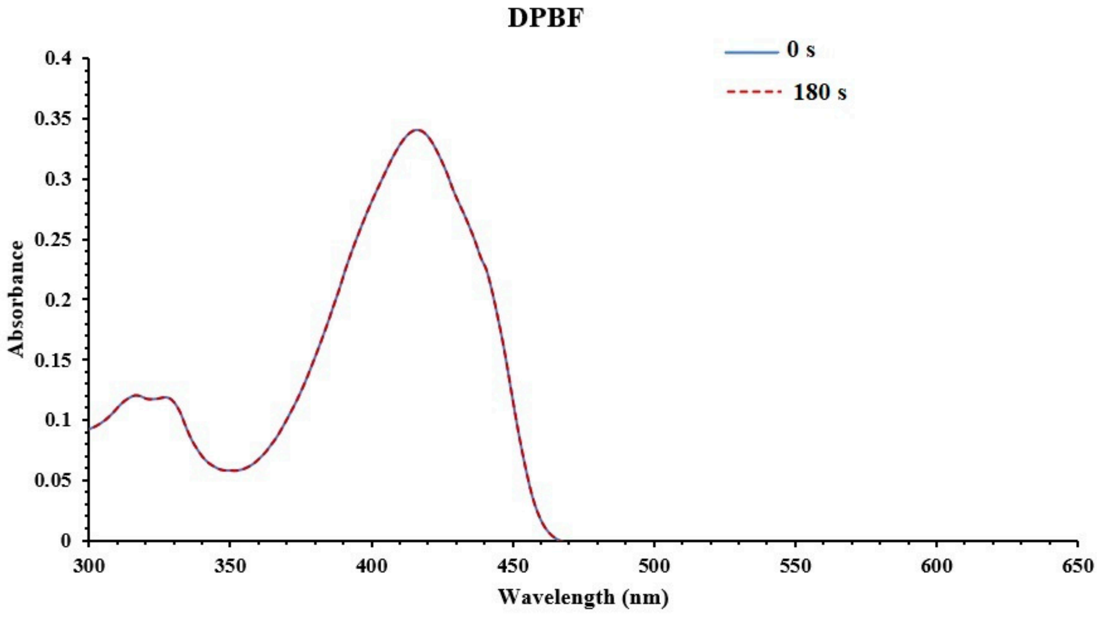

Figure S32. A control experiment was conducted by irradiating a solution of DPBF (12.5 μM) in the absence of photosensitiser with irradiation at 528 nm, in MeCN during 180 s.

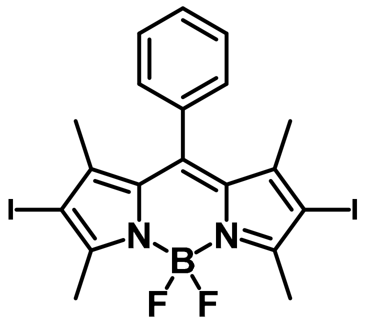

I_2-BDP

Figure S33. Structure of **I_2-BDP** (std)

BSA binding

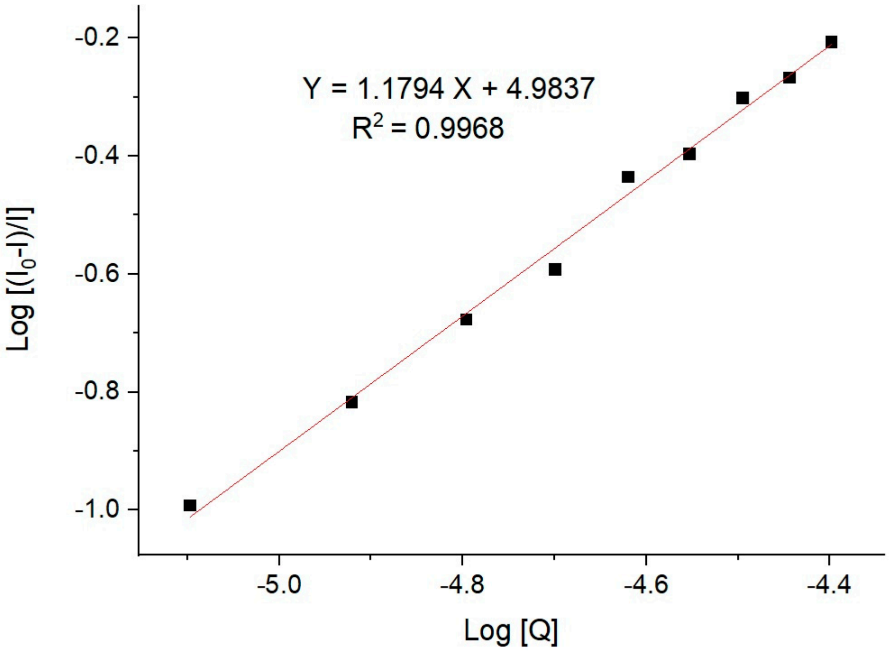

Figure S34. Scatchard plots of the fluorescence titrations of **BDP-2** (0-40 μM) with BSA (50 μM).

Time-correlated Single Photon Counting

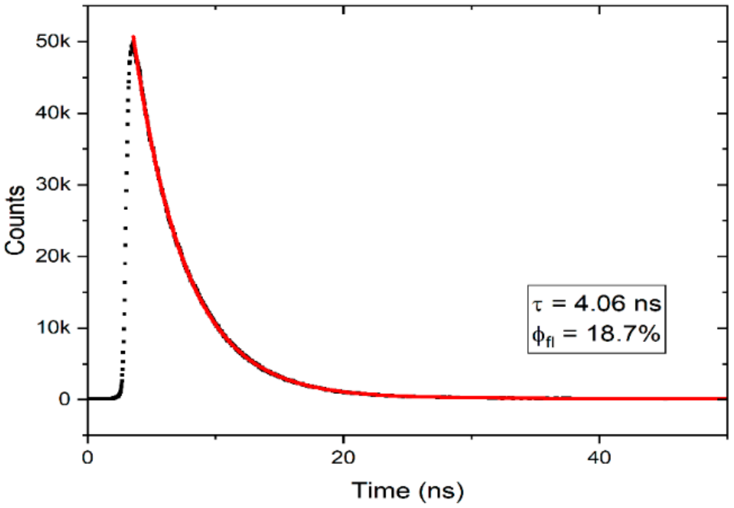

Figure S35. Decay trace for the emission signal of **BDP-2** at 572 nm, in DCM (following excitation at 375 nm). Fluorescence quantum yield determined using the TCSPC integrating sphere.
